# Installation of Copper(I) and Silver(I) Sites into TREN-Based Porous Organic Cages via Postsynthetic Metalation

**DOI:** 10.1021/acs.organomet.4c00247

**Published:** 2024-09-12

**Authors:** Hope A. Silva, Bevan S. Whitehead, Christopher D. Hastings, Chandan Kumar Tiwari, William W. Brennessel, Brandon R. Barnett

**Affiliations:** † Department of Chemistry, University of Rochester, Rochester, New York 14627-0001, United States

## Abstract

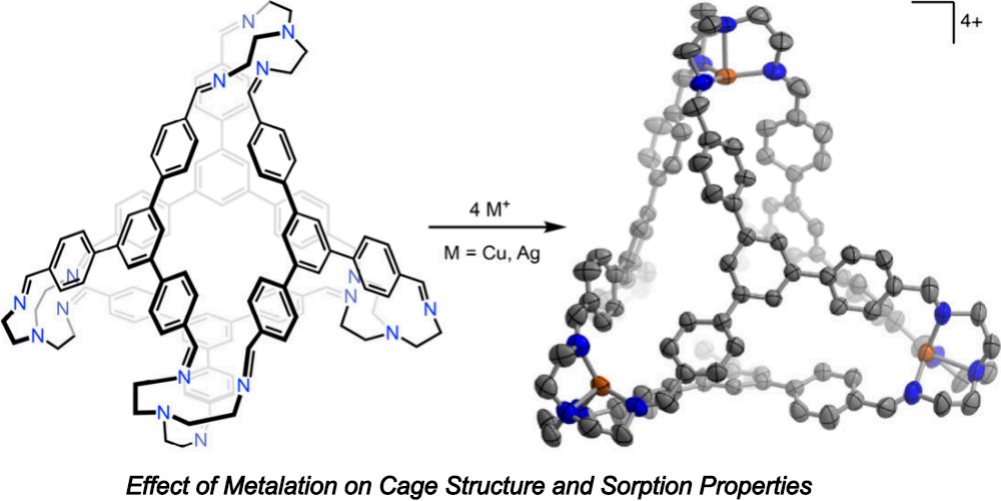

Porous organic cages (POCs) and metal–organic polyhedra (MOPs) function as zero-dimensional porous materials, able to mimic many functions of insoluble framework materials while offering processability advantages. A popular approach to access tailored metal-based motifs in extended network materials is postsynthetic metalation, which allows metal installation to be decoupled from framework assembly. Surprisingly, this approach has only sparingly been reported for molecular porous materials. In this report, we demonstrate postsynthetic metalation of tetrahedral [4 + 4] POCs assembled from tris(2-aminoethyl)amine (TREN) and 1,3,5-tris(4-formylphenyl)benzene. The trigonally symmetric TREN motif is a common chelator in coordination chemistry and, in the POCs explored herein, readily binds copper(I) and silver(I) to form cationic cages bearing discrete mononuclear coordination fragments. Metalation retains cage porosity, allowing us to compare the sorption properties of the parent organic and metalated cages. Interestingly, introduction of copper(I) facilitates activated oxygen chemisorption, demonstrating how targeted metalation can be exploited to tune the sorption characteristics of porous molecular materials.

## Introduction

Hybrid and organic porous framework materials have exploded in popularity owing to their promise in applications that include catalysis, drug delivery, water purification, gas storage, and molecular separations.^1−9^ Inspired by naturally occurring inorganic porous solids such as zeolites and charcoals, metal–organic frameworks (MOFs) and covalent organic frameworks (COFs) stand out for the opportunities they afford to modulate the size, shape, and chemical environment of their internal pores. These crystalline materials are assembled from molecular precursors through the formation of new coordinative (MOFs) or covalent (COFs) linkages. Crucially, the syntheses of crystalline solid-state materials generally rely upon dynamic bond formation processes, allowing for errors to undergo correction under synthetic conditions.^10^ In the case of MOFs, wherein the dynamic connections being formed are new metal–ligand bonds, the use of relatively hard metal ions and donor motifs limits the formation of strong covalent bonds and allows for dynamic bond formation to be accessible at synthetically realistic temperatures. The incorporation of softer and low-valent ions can be carried out through postsynthetic metalation of MOFs or COFs featuring exposed chelating groups^11^ or via cation exchange processes in MOFs,^12^ yielding materials with unique and useful structures.^13,14^

The insoluble nature of solid-state networks presents processability challenges and complicates crystal engineering and phase separation. In contrast, permanently porous molecular cages afford the opportunity to incorporate many favorable characteristics of framework materials into soluble compounds.^15−18^ These zero-dimensional materials generally can be dissolved, allowing for solution-phase processing to be utilized. Porous organic cages (POCs) have been shown to support permanent porosity in both the solid and liquid phases, partly on account of their construction through strong covalent bonds.^16,19−21^ Intriguingly, certain POCs bear free heteroatom-containing motifs that can act as metal-chelating groups, enabling postsynthetic metalation to yield uniform and well-defined metalated cages; however, it is important to note that this strategy has been explored only sparingly in POC systems.^22−24^ In analogy to the metalation of framework materials, postsynthetic cage metalation can potentially lead to cages bearing reactive and low-coordinate metal centers capable of interesting stoichiometric or catalytic reactivity and additionally can alter cage rigidity, charge, and solubility. As metalation is orthogonal to cage construction, it also provides a tractable route to introduce soft, low-valent metal centers that might otherwise be challenging to incorporate.

In coordination chemistry, few chelating platforms can claim histories as rich and extensive as tris(2-aminoethyl)amine (TREN) and its derivatives.^25−33^ Notably, there exist many porous cages that utilize TREN as a tritopic structural building unit, most frequently through combination with partners bearing multiple aldehyde units to generate imine linkages. Such constructs conserve the potential coordinative abilities of the N_4_ pocket, with the imine groups leading to a rather strong-field ligand environment, which should form stable adducts with soft, electron-rich metal ions. Unlike cages bearing ditopic N-based donors, which have the potential to coordinate metals *exo* to the cage interior and facilitate the formation of extended frameworks,^23^ TREN-based chelators should display a strong propensity to form mononuclear coordination environments and retain the molecular nature of the cages. To our knowledge, no examples of metalated TREN-based porous organic cages have been previously reported. We decided to explore the postsynthetic metalation of the [4 + 4] cage (**1**) constructed from TREN and 1,3,5-tris(4-formylphenyl)benzene, which was first reported by Li and co-workers in 2017.^34,35^ This cage has been shown to form clathrates with chlorinated methane solvents, and a close structural analogue was demonstrated to form an inclusion complex with white phosphorus (P_4_). Herein, we show that **1** possesses accessible porosity in the solid state, as does its hydrogenated amine-based congener **2**, which is reported here for the first time. Additionally, we show that both **1** and **2** undergo smooth metalation with copper(I) and silver(I) sources to yield cationic cages **1-M** and **2-M** (M = Cu, Ag) that each bear four discrete [ML_4_]^+^ units while retaining an intrinsic tetrahedral cavity. The effect of metalation on the gas sorption properties of these molecular materials is explored.

## Results/Discussion

**Figure 1:**
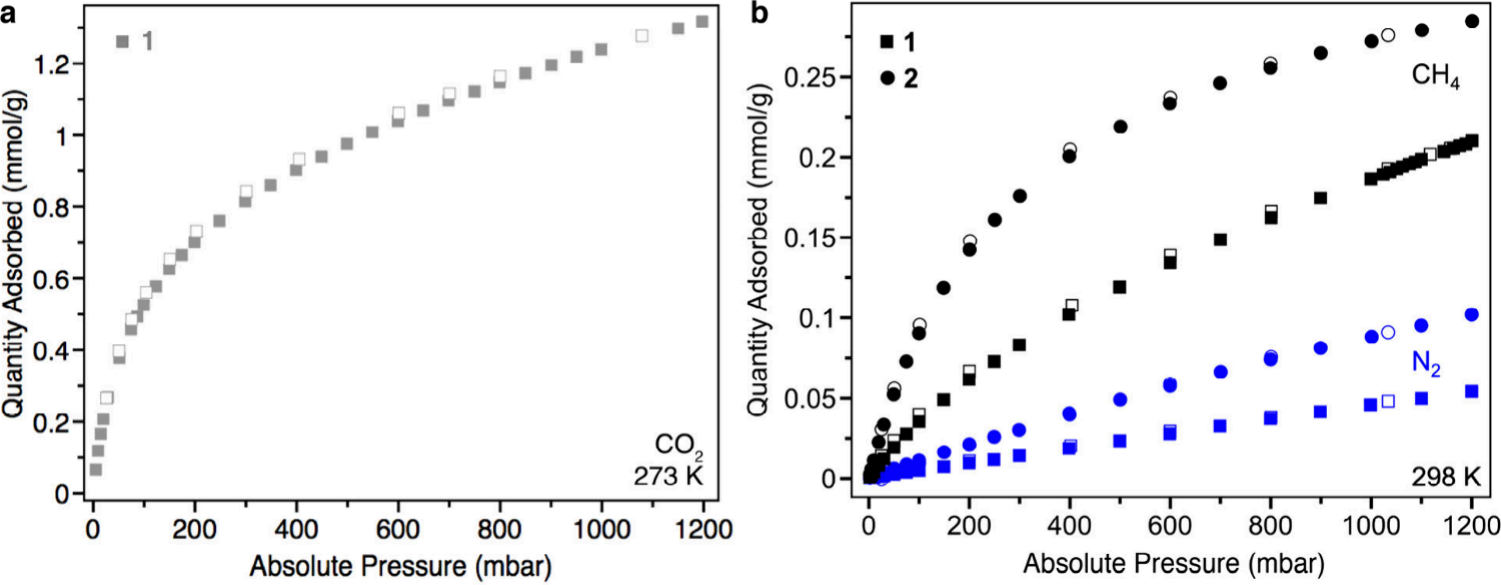
(a) Carbon dioxide sorption isotherm data for **1** at 273 K. (b) Methane and nitrogen sorption isotherm data for **1** and **2** at 298 K. Filled and open symbols represent data points taken during adsorption and desorption, respectively.

The tetrahedral macrocyclic cage **1** contains a central cavity that can accommodate chlorinated methanes.^34^ Self-assembly of **1** in dichloromethane (CH_2_Cl_2_) yields large needle-shaped single crystals of **1**•CH_2_Cl_2_. Heating **1**•CH_2_Cl_2_ liberates CH_2_Cl_2_ to yield the bare cage **1**. Although **1** was reported to be nonporous in the solid state,^34^ our investigation has revealed that measurable uptakes of small gaseous adsorbates can be achieved at ambient temperatures. Carbon dioxide (CO_2_) adsorption at 273 K was chosen as a probe owing to the small and discontiguous pores present in **1**, which kinetically inhibit gas diffusion through the material at cryogenic temperatures.^36^ The Brunauer–Emmett–Teller (BET) surface area obtained from CO_2_ adsorption is 64 m^2^/g, which compares well with other low-porosity molecular cages (Figures 1a and S1). Additionally, **1** adsorbs methane (CH_4_) with a Langmuir isotherm profile at 298 K, achieving an uptake of 0.22 mmol/g at 1.2 bar (Figure 1b), which corresponds to 0.42 equiv of CH_4_ per cage. In contrast, adsorption of nitrogen occurs to only a very small extent at this temperature.

The use of Ideal Adsorbed Solution Theory^37^ results in a calculated selectivity of 7.1 for a 50:50 mixture of CH_4_ and N_2_ at 298 K and 1 bar total pressure (Figure S7). This value comes with the caveat that nitrogen uptakes are quite low, and numerical isotherm fitting equations that do not extrapolate well at higher pressures can induce significant errors into the determined selectivity value. We have found the N_2_ isotherm data to be well modeled by a single-site Langmuir–Freundlich fit. By constraining the saturation capacity value for this site to correspond to a loading of 1.0 N_2_ per cage (0.52 mmol/g) and ensuring this fit is of high quality within the experimental range of pressures, we have sought to minimize such errors to the greatest extent possible. It should also be noted that adsorption in porous cage compounds can occur at either intrinsic or extrinsic voids,^16^ and the higher polarizability of CH_4_ typically results in modestly higher adsorbed quantities compared to N_2_ at a given pressure and temperature. However, we suggest the substantial differences in uptakes between these adsorbates seen in **1** reflect preferential adsorption of methane at the intrinsic cage cavity on account of their complementary tetrahedral shapes.

**Figure 2:**
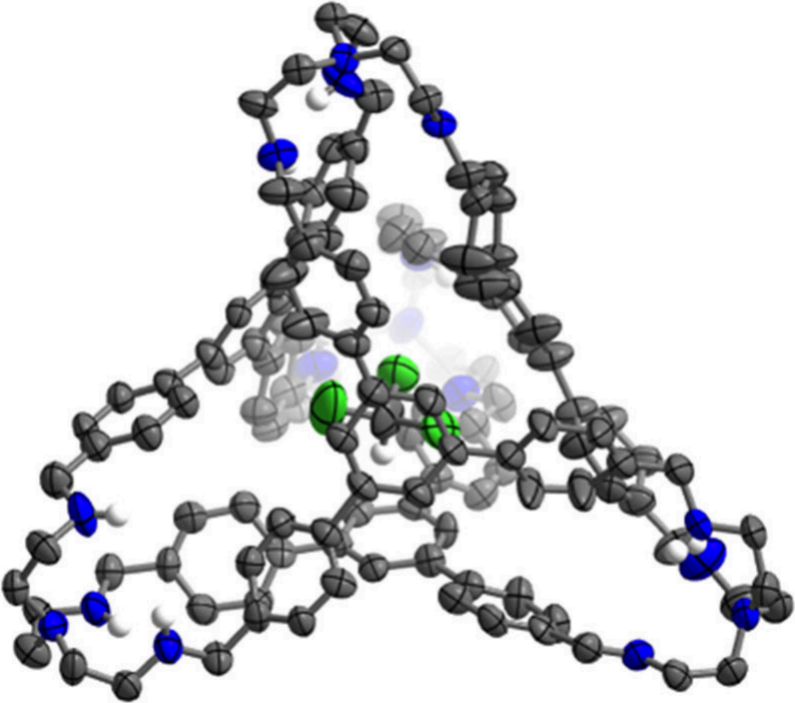
Solid-state structure of **2**•CHCl_3_, showing one of the two cages present in the asymmetric unit. Nonencapsulated chloroform molecules and all cage C–*H* hydrogen atoms have been omitted for clarity. Thermal ellipsoids are depicted at a 50% probability level.

**Scheme 1:**
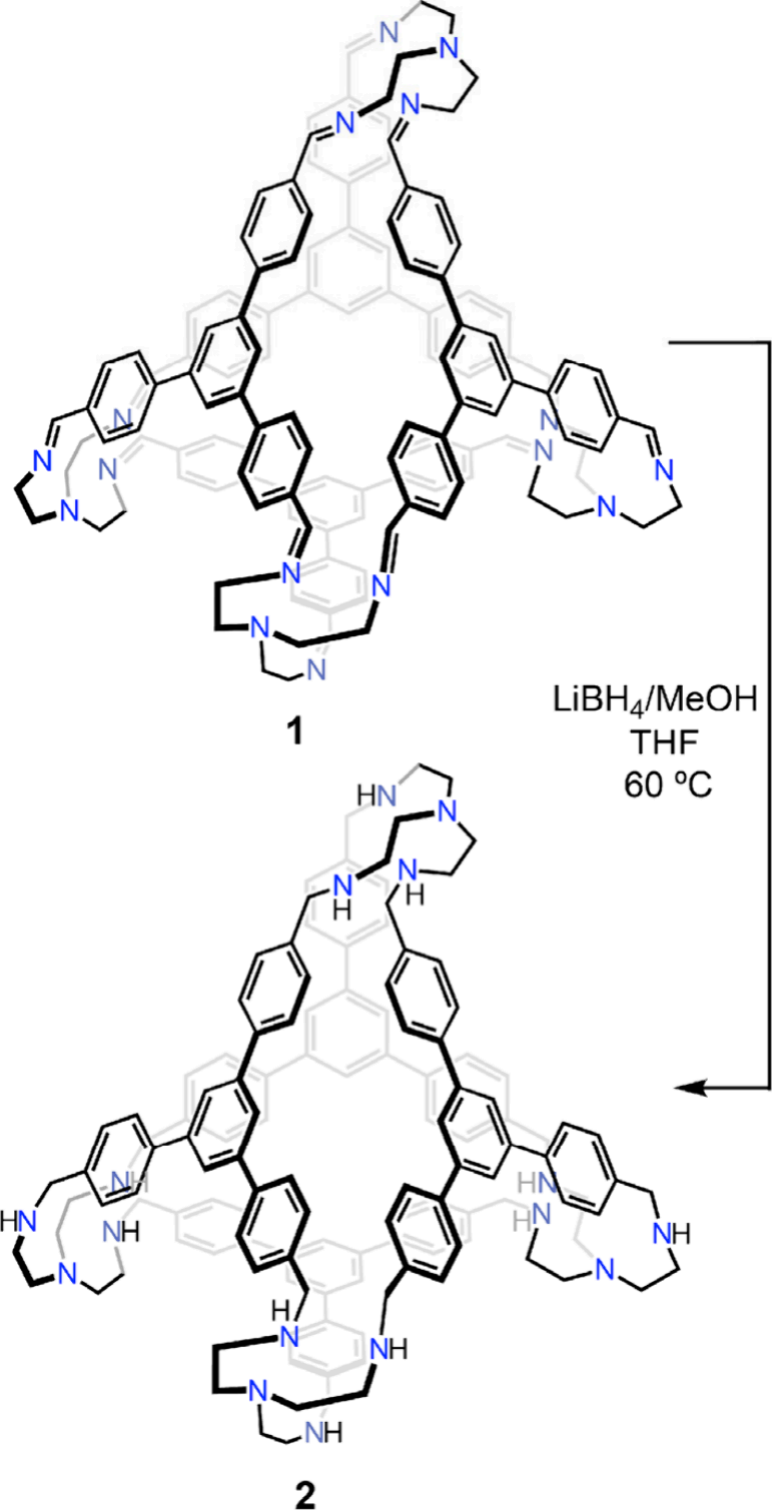
Hydrogenation of the Imine Linkages in **1** to Secondary Amines in **2**

So as to modify the electronic makeup of the TREN-based metal-binding pockets, the imine linkages of **1** were hydrogenated using lithium borohydride to produce the reduced macrocycle **2** as a colorless solid (Scheme 1). Reduction of imine-based POCs to yield secondary amine groups has ample precedent, with the resulting cages generally displaying enhanced flexibilities and hydrolytic stabilities.^38,39^ Reduction of all 12 imine functional groups is evident from the disappearance of the N=C–*H* resonance in the ^1^H NMR spectrum, coupled with the appearance of two new multiplets at 1.37 and 3.75 ppm attributable to N–*H* and benzylic methylene (−C*H*_2_−) motifs, respectively (Figure S24). Imine hydrogenation leads to the disappearance of the feature at 1645 cm^–1^ in the infrared spectrum of **1**, corresponding to eradication of the C=N double bond (Figure S16). Additionally, a band at 3281 cm^–1^ grows in for **2** that is consistent with the formation of N–H bonds (Figure S16). A series of acidic and basic washes was employed following reduction to ensure that no boron-containing byproducts remained encapsulated within the cage interior. Recrystallization from a CHCl_3_ solution layered with diethyl ether yields colorless crystals of **2**•CHCl_3_, wherein one disordered chloroform guest resides within the intrinsic cavity of the cage (Figure 2).

A common trait of permanently porous materials is structural rigidity, which decreases the proclivity for pore collapse upon guest removal. As imine hydrogenation replaces C=N double bonds with more conformationally flexible single bonds, it might be expected that **2** would lose the ability to adsorb gases in the solid state. However, **2** indeed retains permanent porosity and displays increased uptakes of both CH_4_ and N_2_ at 298 K in comparison to **1**, achieving a CH_4_ loading at 1200 mbar that corresponds to 0.55 equiv per cage (Figure 1). We speculate that hydrogenation imbues **2** with a small amount of flexibility that yields a cavity with some guest-adaptive properties, allowing for increased host–guest interactions upon intercalation. The conservation of selectivity for adsorption of CH_4_ over N_2_ is again consistent with the intrinsic cavity of **2** serving as the primary site for gas uptake. Compared to **1**, the selectivity determined from IAST (50:50 mixture at 298 K and 1 bar) increases from 7.1 to 9.4 (Figure S7).^40^ We note that **2** displays a selectivity that compares well with many of the top-performing framework materials for the separation of these gases, which is a critical component of natural gas purification.^41,42^ However, the modest uptakes seen here would render its implementation for this application impractical. Surface area measurements using CO_2_ were hampered by sluggish sorption kinetics as evidenced by hysteresis between the adsorption and desorption sweeps (Figure S2). Contributing factors may include intercage hydrogen bonding (which could inhibit the dynamic processes necessary for diffusion between discontiguous pores) and slow chemisorption at the secondary amine sites.^43^ Although the CO_2_ isotherm for **2** is not suitable for determining a BET surface area, the measured uptakes are comparable to those seen for **1**.

**Figure 3:**
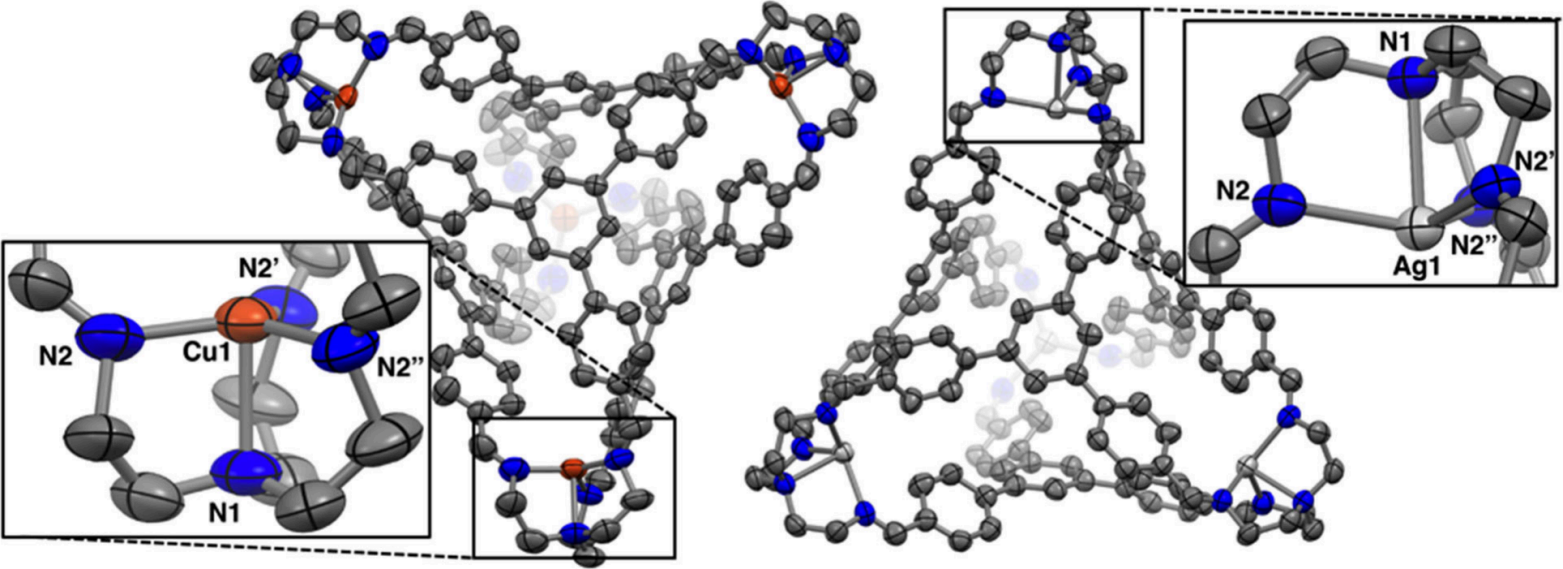
Solid-state structures of the tetracationic cages **1-Cu** (left) and **1-Ag** (right). The intrinsic cavities remain free of either anions or solvent molecules for both structures. Hydrogen atoms, triflate counteranions, and cocrystallized solvent molecules are omitted for clarity. Thermal ellipsoids are depicted at a 30% (**1-Cu**) or 50% (**1-Ag**) probability level. Selected bond distances (Å) and angles (deg) for **1-Cu**: Cu1–N1 = 2.202(6); Cu1–N2 = 2.022(5); N1–Cu1–N2 = 85.12(11). Selected bond distances (Å) and angles (deg) for **1-Ag**: Ag1–N1 = 2.390(5); Ag1–N2 = 2.269(3); N1–Ag1–N2 = 77.97(7).

**Scheme 2:**
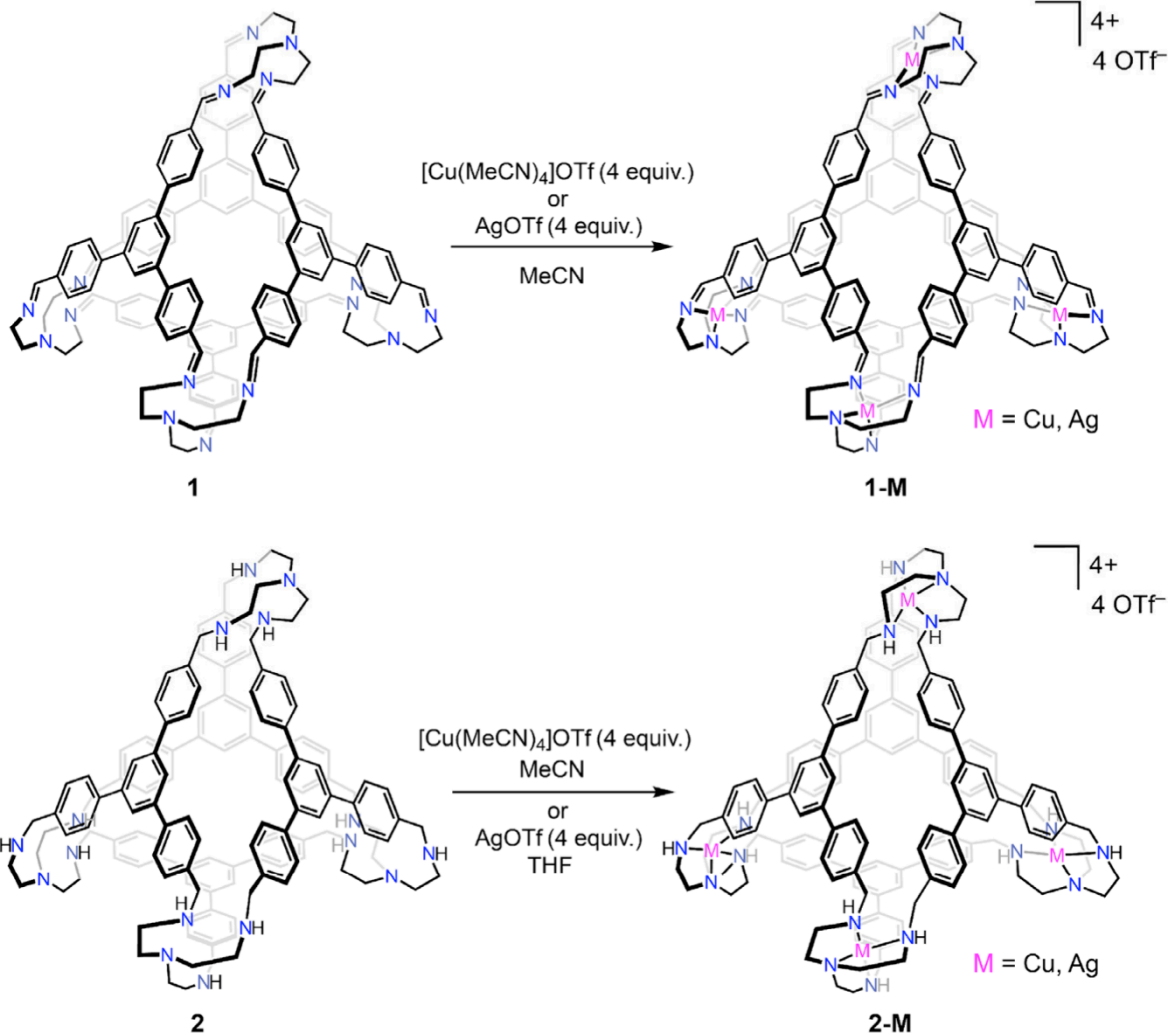
Metalation of **1** (top) and **2** (bottom) with Both Copper(I) and Silver(I)

As evidenced from their solid-state structures, both **1** and **2** contain tripodal tetra-aza pockets that appear well-suited to chelate a metal center. Indeed, exposure of these cages to either [Cu(NCMe)_4_]OTf or AgOTf (OTf^–^ = trifluoromethanesulfonate = triflate) results in clean metalation at all four binding pockets to yield **1-M** and **2-M** (M = Cu, Ag; Scheme 2). Single crystals of **1-Cu** and **1-Ag** suitable for X-ray diffraction were grown by allowing diethyl ether to diffuse into an *N,N*-dimethylformamide (DMF) solution (Figure 3). The cages adopt isomorphous packing arrangements in the *P*-43*n* space group, with the asymmetric unit consisting of only one metal site and one arm of the TREN unit. Accordingly, all metal sites are equivalent and display crystallographically imposed threefold rotational symmetry. The coordination geometries observed are best described as trigonal monopyramidal, with τ_4_ values^44^ of 0.86 for **1-Cu** and 0.91 for **1-Ag**. Although partial occupation of the intrinsic void with a disordered solvent molecule appears to occur (see Section S4 of the Supporting Information), the accessible porosity observed for these cages (see below) indicates that any intracavity guests can be removed upon heating *in vacuo*. The triflate anions are highly disordered and could not be appropriately modeled (see Section 4 of the Supporting Information). However, the number of intracavity electrons found by the Platon program SQUEEZE^45^ is significantly lower than present in 1 equiv of OTf^–^. Additionally, ^19^F NMR spectra indicate that only one triflate environment is present in solution (Figures S27 and S33), suggesting that all counterions remain *exo* to the cages.

**Figure 4:**
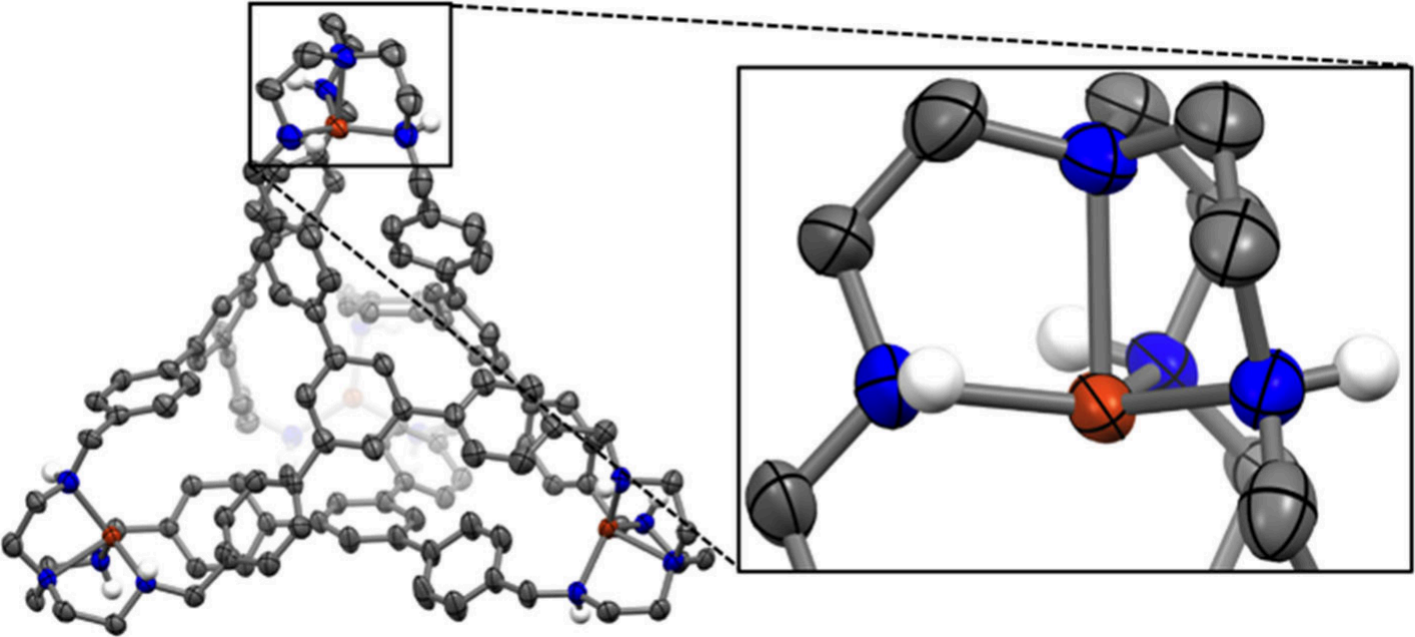
Solid-state structure of the tetracationic cage **2-Cu**•DMF. The intracavity DMF guest is omitted for clarity. Also omitted are all carbon-bound hydrogen atoms, non-coordinating triflate counteranions, and co-crystallized solvent molecules. The asymmetric unit contains one complete Cu_4_ cage. Thermal ellipsoids are depicted at a 50% probability level. Axial Cu–N bond distances range from 2.220(3) to 2.255(3) Å. Equatorial Cu–N bond distances range from 2.060(4) to 2.129(4) Å. N_ax_–Cu–N_eq_ angles range from 82.07(14) to 86.21(16)°.

Metalation of **2** was carried out using similar synthetic procedures as for **1**, and the copper congener **2-Cu** was characterized via single-crystal X-ray diffraction measurements using crystals grown from diethyl ether diffusion into a DMF solution (Figure 4). This structure crystallizes in the orthorhombic space group *Pbca*, with the asymmetric unit consisting of one complete formula unit of the tetracationic cage and four triflate anions. The copper coordination environments are again best described as trigonal monopyramidal (τ_4_ values between 0.81 and 0.85; see Table S9).^44^ The Cu–N_eq_ bond distances (range of 2.060(4)–2.129(4) Å) are elongated in comparison to those in **1-Cu** (2.022(5) Å), likely on account of the ability of the imine groups in **1-Cu** to act as both σ-donors and π-acids, whereas the secondary amine donors in **2-Cu** functions primarily as σ-donors. An ordered molecule of DMF resides within the cage as an intracavity guest, while all four trifluoromethanesulfonate anions are located outside of the cage interior (Figure S22). Metalation of **2** with AgOTf analogously furnishes **2-Ag**, which was isolated and characterized via NMR spectroscopy and elemental analysis. As seen for **2-Cu,** the ^1^H NMR resonances corresponding to the secondary amine N–*H* protons in **2-Ag** shift downfield compared to those in **2**, while the observation of only one peak in the ^19^F NMR spectrum intimates that all triflate anions reside outside of the cages (Figures S35–36). Unfortunately, formation of darkly colored inorganic byproducts (presumably consisting of metallic silver) during the synthesis complicated purification and consistently led to low isolated yields. Accordingly, gas sorption measurements on **2-Ag** were not undertaken.

**Figure 5:**
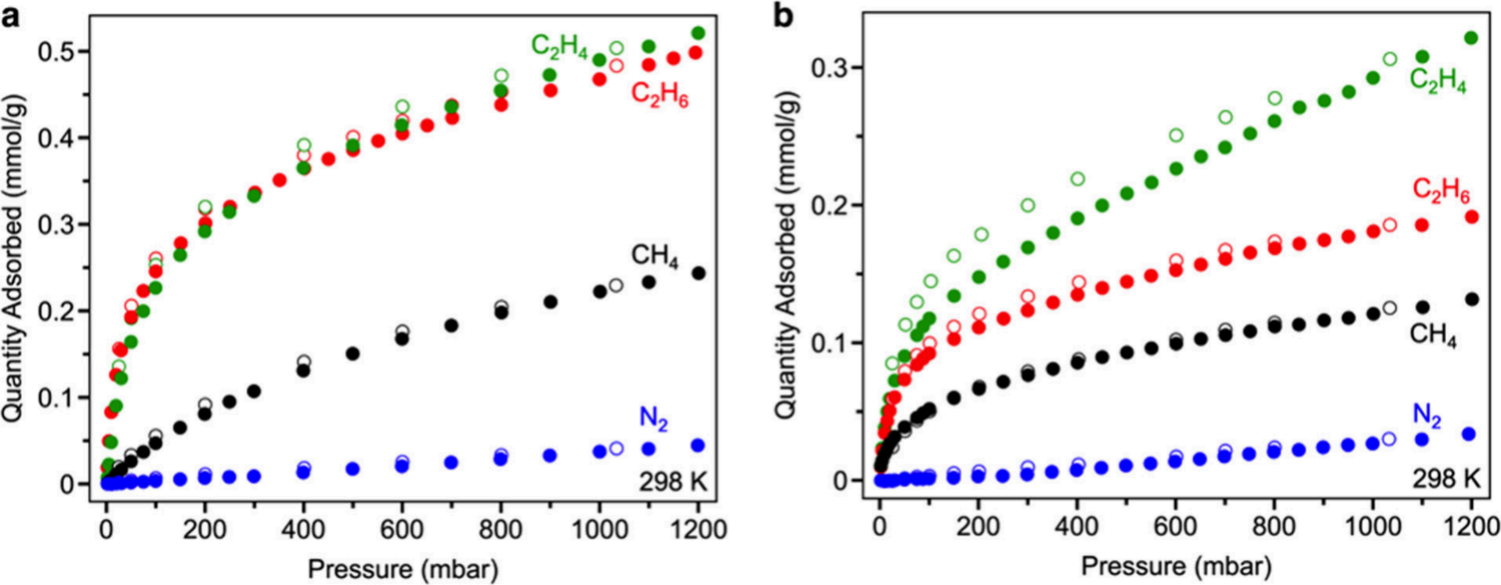
Gas sorption isotherms for **1-Cu** (a) and **2-Cu** (b) at 298 K. Filled and open circles correspond to data points collected during adsorption and desorption, respectively.

The uptakes of CO_2_ at 273 K for the metalated cages are lower than for their nonmetalated congeners, a fact that is due largely to the increases in molecular weights.^46^ In comparing the sorption properties of **1-Cu** and **2-Cu**, we observe canonical Type I methane isotherms at 298 K that mirror those obtained for the nonmetalated cages, and selectivity for adsorption of methane over nitrogen is clearly maintained (Figure 5). The presence of nonporous triflate anions throughout the lattice slows diffusion and renders the attainment of equilibration more challenging. In particular, the nitrogen isotherms deviate from Langmuir-type behavior owing to very slow diffusion and low uptake values. We have omitted the determination of IAST selectivities for these metalated macrocycles, as it appears unlikely that the nitrogen uptake values reflect truly equilibrated data points.

The trigonal monopyramidal coordination geometries apparent within **1-M** and **2-M** led us to inquire whether the open coordination sites could be accessible to adsorbates. This notion was probed by measuring isotherms of both ethylene and ethane for **1-Cu**, **2-Cu**, and **1-Ag**. While most adsorbents lacking open metal sites display similar affinities for these C_2_ gases owing to their similar sizes and polarizabilities, the presence of even weakly Lewis acidic metal sites can lead to significant selectivities for the olefin.^47^ All three metalated cages uptake significant amounts of ethylene and ethane at 298 K, and the isotherms for both gases in a given cage closely overlie at low pressures, where uptake capacities correlate most strongly with adsorption enthalpy (Figures 5 and S14). This observation suggests that the open metal sites are not relevant in C_2_ hydrocarbon adsorption. For **2-Cu**, higher uptakes for ethylene in comparison to ethane are seen at higher pressures. We attribute this observation to the smaller size of ethylene, which may both facilitate faster diffusion and allow for access to voids that are less accessible to larger ethane.^1^

The 18-electron configurations of the metal sites should render them as only weakly Lewis acidic. However, we also note that the solid-state structure of **1-Cu** reveals that each aryl ring orients in a way that obscures most of the area surrounding the open axial sites at the metal centers (Figure S19). This arrangement minimizes the dihedral angle between the C=N imine bond and the aryl ring and maximizes conjugation of the π-systems. As a result, the *ortho*-C–*H* bonds point directly toward the metal sites. Owing to their close proximities and the large M···H–C angles (141°),^48^ these interactions are best described as anagostic in nature.^49^ An analogous conformation is seen in the solid-state structure of **1-Ag** (Figure S20). Eradication of the C=N double bonds in **2** increases conformational flexibility in the secondary coordination sphere. Two of the three surrounding aryl rings point their faces toward the axial coordination site, while the third ring points its edge inward (Figure S21). On account of this “gearing” arrangement,^50,51^ the cuprous sites in **2-Cu** remain isolated from the intrinsic cavity, although they appear perhaps more accessible than those in rigid **1-Cu**.

**Figure 6:**
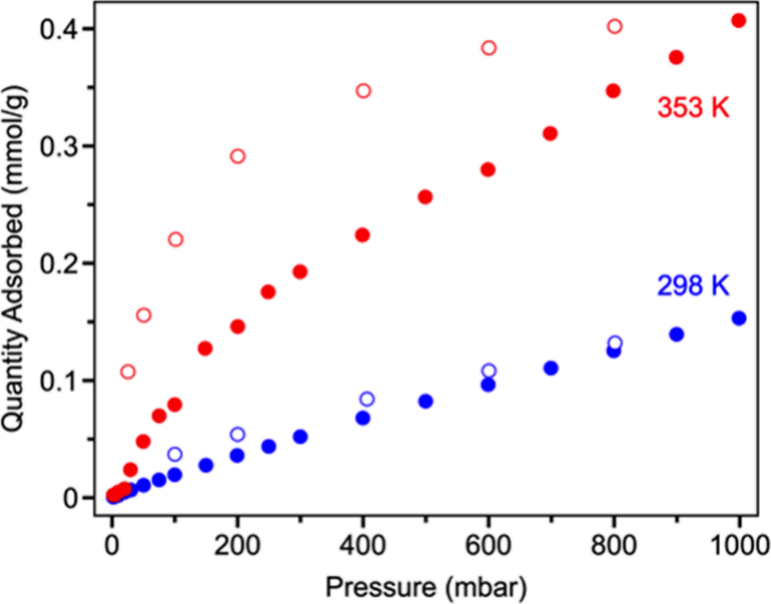
Oxygen isotherms for **2-Cu** at 298 and 353 K. Filled and open circles correspond to data points collected during adsorption and desorption, respectively.

Trigonal monopyramidal cuprous species can react with dioxygen to form (often transient) adducts best described as copper(II) superoxides.^52−57^ We reasoned that the flexibility of **2-Cu** may allow for O_2_ to access the copper centers and undergo chemisorption. Equilibrium isotherm measurements at 298 K reveal negligible uptake, suggesting a large kinetic barrier to chemisorption. Notably, however, raising the temperature to 353 K reveals significant increases in capacity compared to 298 K, reaching an uptake of 0.41 mmol/g (1.2 equiv of O_2_ per cage; Figure 6). A large hysteresis between adsorption and desorption is consistent with a slow chemisorption process,^58^ as is the observation that subsequent 353 K O_2_ isotherms fail to recover most of capacity seen in the initial measurement (Figure S9). Additionally, we note that a comparison between N_2_ isotherms collected at 298 and 353 K reveals negligible uptakes at both temperatures (Figure S15). Given the similar kinetic diameters of O_2_ and N_2_,^1^ the increased O_2_ uptakes at high temperature cannot be simply ascribed to faster gas diffusion through the solid material.

**Figure 7:**
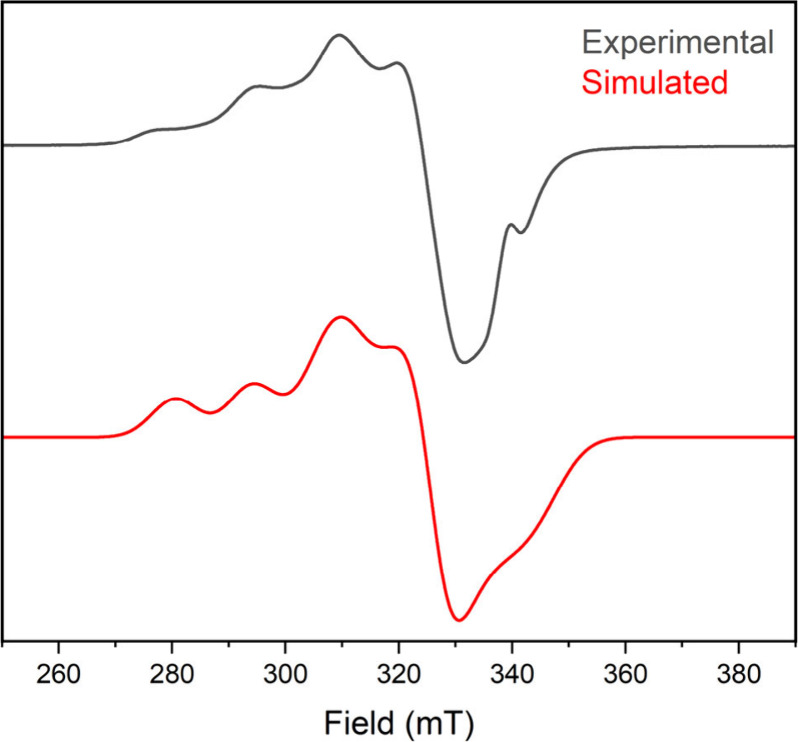
Experimental (gray) and simulated (red) X-band EPR spectrum of **2-Cu** following oxygen chemisorption at 353 K. Experimental parameters: *T* = 10 K; microwave frequency = 9.3931 GHz; power = 1.0 mW; modulation amplitude = 1.0 mT. Simulation parameters: *g* = 2.231, 2.105, 1.990; *A*(^63/65^Cu) = 415.12, 100.86, 135.00 MHz; llwp = 6.9478 mT; *g* strain = 0.036, 0.0045, 0.0499; *A* strain = 0, 0.0747, 0.036 MHz.

Oxygen chemisorption is also evident from spectroscopic comparison of **2-Cu** before and after high-temperature O_2_ exposure. X-band electron paramagnetic resonance (EPR) spectra of solid **2-Cu** following an 80 °C oxygen isotherm measurement (Figure 7) show a new feature that was simulated with rhombic symmetry (*g* = 2.231, 2.105, 1.990), indicating oxidation of some copper(I) centers to copper(II). Comparative electronic spectra show the appearance of new features at 400 and 665 nm (Figure S18). We suggest that initial inner-sphere coordination forms an incipient copper(II) superoxide that reacts further via homolysis of a benzylic C–H bond in an analogous fashion to Reinaud’s calix[6]tren copper system.^57^ Indeed, our EPR and electronic spectra are in excellent agreement with the formation of trigonal bipyramidal cupric centers.^57,59^ While conclusive identification of the O_2_ chemisorption product has proven elusive, we note that transmission infrared spectra show no evidence of O–H or C=O bonds (Figure S17); we accordingly surmise that this species does not contain a hydroperoxo or hydroxyl ligand, nor does it contain an oxidized organic scaffold bearing a hydroxide or amide group.

Although imine cage **1-Cu** also displays an increase in O_2_ uptake at 353 K compared to 298 K, this increase is rather modest (Figure S8). This may reflect the decreased steric accessibility of the metal sites in **1-Cu** compared to those in **2-Cu** but could also be connected to the π-accepting properties of the imine donors, which would render the formal reduction of O_2_ by **1-Cu** more challenging than for **2-Cu**.

## Conclusion

Postsynthetic metalation of the TREN-based [4 + 4] cages **1** and **2** provides a route to install low-valent metal sites without significantly altering the cage shape or facilitating the formation of extended networks. This effort adds to the small number of previous works reporting the metalation of POCs and demonstrates a route to install robust and well-defined mononuclear metal sites capable of substrate chemisorption. The trigonal monopyramidal Cu^+^ and Ag^+^ centers remain largely isolated from the cage interior, and accordingly, significant kinetic barriers must be overcome for adsorbates to access these sites. Future efforts will focus on optimizing cage design to improve the accessibility of metal sites to intracage guests.

## Experimental Section

### General Considerations

Unless otherwise stated, all reagents were purchased from commercial sources and used as received or purified using standard procedures.^60^ Molecular sieves and Celite were separately preactivated in a 180 °C oven overnight, then transferred into a round-bottom flask and heated under vacuum (*P* < 100 mTorr) at a temperature in excess of 200 °C for at least 12 h, and then stored in the glovebox. Tetrahydrofuran (THF), diethyl ether (Et_2_O), acetonitrile (MeCN), and *N,N*-dimethylformamide (DMF) were dried and deoxygenated using a Pure Process Technologies solvent purification system. Dimethylsulfoxide-*d*_6_ (DMSO-*d*_6_) was dried overnight on activated 4 Å sieves, transferred via cannula to a Strauss-style flask, deoxygenated by sparging with N_2_ (1 h), and stored in the glovebox over freshly activated 3 Å sieves for at least 1 day prior to use. All other solvents were used as received from commercial sources. The imine-linked based macrocyclic cage **1** was synthesized according to a published procedure.^34^ Solution ^1^H, ^13^C{^1^H}, and ^19^F nuclear magnetic resonance (NMR) spectra were recorded on a Bruker DPX-400, Bruker DPX-500, JEOL-500, or JEOL-400 spectrometer locked on the signal of deuterated solvents. ^1^H and ^13^C{^1^H} chemical shifts are reported in parts per million relative to SiMe_4_ (^1^H and ^13^C δ = 0.00 ppm) with reference to residual solvent resonances. ^19^F chemical shifts are reported relative to CFCl_3_ (δ = 0.00 ppm). Combustion analysis was performed at the CENTC Elemental Analysis Facility (University of Rochester) using a PerkinElmer 2400 Series II analyzer. X-band EPR measurements were carried out on a Bruker EMXplus spectrometer. Samples were prepared as finely ground powders in a glovebox, flash-cooled in liquid nitrogen within quartz sample tubes, and loaded into the spectrometer. Simulation of EPR spectra was performed using the program *EasySpin*.^61^

### Synthesis of **2**

In a nitrogen-filled glovebox, a 50 mL Schlenk flask was charged with **1** (0.360 g, 0.187 mmol) and lithium borohydride (0.250 g, 11.5 mmol, 62 equiv). THF (35 mL) was added, and the Schlenk flask was placed on a Schlenk line under N_2_ outside of the glovebox. Under active N_2_, methanol (0.595 mL, 14.7 mmol, 79 equiv) was added via syringe, and the reaction was allowed to stir at 60 °C for 48 h. After cooling to room temperature, while still under active N_2_, water (20 mL) was added via syringe, and the mixture was allowed to stir overnight. After the mixture was dried *in vacuo*, the resulting crude white solid was combined with 1 M HCl (40 mL) and EtOH (90 mL) in a 250 mL round-bottomed flask. The suspension was stirred overnight and then filtered through a medium-porosity fritted funnel. The crude solid was washed with H_2_O and Et_2_O and then was combined with 1 M KOH (40 mL) and EtOH (90 mL) in a 250 mL round-bottomed flask. The suspension was stirred overnight and then filtered through a medium-porosity fritted funnel. The crude solid was washed with H_2_O and then was combined with H_2_O (150 mL) in a 250 mL round-bottomed flask. The suspension was allowed to stir at 80 °C overnight and then filtered through a medium-porosity fritted funnel. To the filter cake, CHCl_3_ was added, which dissolves the desired product while leaving behind some insoluble impurities. The resulting filtrate was dried *in vacuo* to yield **2** as a colorless solid. Yield: 0.305 g, 0.157 mmol, 84%. ^1^H NMR (500 MHz, CDCl_3_): δ = 7.15 (s, 48H), 7.12 (s, 12H), 3.82 (s, 24 H), 2.93 (m, 12H), 2.80 (m, 12H), 2.73 (m, 12H), 2.68 (m, 12H), 1.25 (s, 12H). ^1^H NMR (500 MHz, C_6_D_6_) δ 7.47 (s, 12H), 7.36 (d, *J* = 8.0 Hz, 24H), 7.31 (t, *J* = 6.6 Hz, 30H), 7.00 (s, 6H), 3.74 (s, 24H), 2.67 (br s, 24 H), 2.50 (br s, 24H). ^13^C{^1^H} NMR (101 MHz, CDCl_3_): δ = 141.88, 139.87, 139.65, 127.66, 127.16, 124.73, 55.03, 54.13, 48.48 ppm. Calculated elemental analysis for C_133_H_145_N_16_Cl_3_ (**2**•1.0 CHCl_3_): C, 77.02%; H, 7.05%; N, 10.81%. Found: C, 77.00%; H, 7.29%; N, 10.81%.

### Synthesis of **1-Cu**

In a nitrogen-filled glovebox, a scintillation vial was charged with **1** (0.070 g, 0.036 mmol) and tetrakis(acetonitrile)copper(I) triflate (0.743 g, 0.197 mmol, 5.5 equiv). Acetonitrile (15 mL) was added, and the orange suspension was stirred for 16 h. Outside of the box, acetonitrile was added to the suspension until the solids were dissolved. Subsequently, Et_2_O was added to precipitate the desired complex. The suspension was filtered through a fine-porosity fritted funnel and washed with excess Et_2_O. The orange residue was collected and dissolved in DMF. Et_2_O was vapor diffused into the orange solution, yielding the desired complex in the form of orange crystals. The crystals were harvested, triturated with warm Et_2_O for several minutes, and isolated by decantation, yielding **1-Cu** after drying *in vacuo*. Yield: 0.043 g, 0.037 mmol, 42%. ^1^H NMR (500 MHz, DMSO-*d*_6_): δ = 8.79 (s, 12H), 8.43 (d, *J* = 8.1 Hz, 24H), 7.38 (s, 12H), 7.25 (d, *J* = 8.0 Hz, 24H), 4.16 (br s, 12H), 3.73 (d, *J* = 12.9 Hz, 12H), 3.20 (br s, 24H). ^13^C{^1^H} NMR (126 MHz, DMSO-*d*_6_): δ = 161.90, 142.24, 140.07, 132.93, 129.21, 126.35, 123.96, 59.87, 52.27 ppm. ^19^F NMR (470 MHz, DMSO-*d*_6_): δ = −78.0 (s) ppm. Calculated elemental analysis for C_139_H_127_N_17_O_13_F_12_S_4_Cu_4_ (**1-Cu**•1.0 DMF): C, 58.50%; H, 4.49%; N, 8.34%. Found: C, 58.13%; H, 4.42%; N, 8.33%.

### Synthesis of **1-Ag**

In a nitrogen-filled glovebox, a scintillation vial was charged with **1** (0.175 g, 0.091 mmol) and silver(I) trifluoromethanesulfonate (0.134 g, 0.521 mmol, 5.8 equiv). Acetonitrile (15 mL) was added, and the colorless solution was stirred for 27 h. After the acetonitrile was removed *in vacuo*, the colorless solid was triturated with tetrahydrofuran (THF), and the suspension was then filtered through a fine-porosity fritted funnel and washed with excess THF. The resulting white residue was then dissolved in acetonitrile, and Et_2_O was vapor diffused into the colorless solution, yielding the desired complex in the form of colorless crystals. The crystals were harvested, yielding **1-Ag** after drying *in vacuo*. Yield: 0.148 g, 0.050 mmol, 55%. ^1^H NMR (500 MHz, DMSO-*d*_6_): δ = 8.52 (br s, 12H), 7.81 (d, *J* = 7.9 Hz, 24H), 7.48–7.41 (m, 36H), 3.87 (m, 12H), 3.71 (d, *J* = 12.5 Hz, 12H), 3.12 (t, *J* = 12.3 Hz, 12H), 2.83 (d, *J* = 12.5 Hz, 12H). ^13^C{^1^H} NMR (126 MHz, DMSO-*d*_6_): δ = 162.78, 143.38, 141.42, 134.12, 127.78, 127.72, 125.34, 58.22, 53.60. ^19^F NMR (470 MHz, DMSO-*d*_6_): δ = −78.0 (s) ppm. Calculated elemental analysis for C_136_H_120_N_16_O_12_F_12_S_4_Ag_4_ (**1-Ag**): C, 55.22%; H, 4.09%; N, 7.58%. Found: C, 54.76%; H, 4.15%; N, 8.10%.

### Synthesis of **2-Cu**

In a nitrogen-filled glovebox, a scintillation vial was charged with **2** (0.681 g, 0.348 mmol) and tetrakis(acetonitrile)copper(I) triflate (0.658 g, 1.750 mmol, 5.0 equiv). Acetonitrile (15 mL) was added, and the white suspension was stirred for 24 h. The suspension was then filtered through a fine-porosity fritted funnel and washed with excess acetonitrile. The resulting white residue was then dissolved in DMF, and Et_2_O was added to the filtrate to precipitate the desired complex. The suspension was filtered through a fine-porosity fritted funnel and washed with excess Et_2_O. The white residue was triturated in warm Et_2_O and then isolated by decantation, yielding **2-Cu** as a white solid after drying *in vacuo*. Yield: 0.140 g, 0.050 mmol, 14%. ^1^H NMR (500 MHz, DMSO-*d*_6_): δ = 7.29 (br s, 48H), 7.06 (br s, 12H), 4.17 (br s, 12H), 3.88 (br s, 12H), 3.64 (br s, 12H), 3.04 (br s, 12H), 2.78 (br s, 36H). ^13^C{^1^H} NMR (101 MHz, DMSO-*d*_6_): δ = 141.35, 139.43, 138.10, 127.83, 126.68, 124.60, 54.75, 51.42, 49.98. ^19^F NMR (470 MHz, DMSO-*d*_6_): δ = −78.0 (s) ppm. Calculated elemental analysis for C_136_H_144_N_16_O_12_F_12_S_4_Cu_4_ (**2-Cu**): C, 58.23%; H, 5.17%; N, 7.99%. Found: C, 57.84%; H, 5.31%; N, 7.87%.

### Synthesis of **2-Ag**

In a nitrogen-filled glovebox, a scintillation vial was charged with **2** (0.205 g, 0.105 mmol) and silver(I) trifluoromethanesulfonate (0.160 g, 0.623 mmol, 5.94 equiv). THF (15 mL) was added, and the orange solution was stirred for 1 h. The dark suspension was filtered through a fine-porosity fritted funnel and washed with excess THF. To the filter cake, acetonitrile (10 mL) was added, which dissolves the desired product while leaving behind some insoluble impurities. Et_2_O was vapor diffused into the light yellow solution, yielding the desired complex in the form of colorless crystals. The crystals were harvested, yielding **2-Ag** after drying *in vacuo*. Yield: 0.020 g, 0.007 mmol, 6%. ^1^H NMR (500 MHz, DMSO-*d*_6_): δ = 7.31 (br s, 24H), 7.23 (br s, 24H), 7.01 (br s, 12H), 3.80 (br s, 24H), 3.48 (br s, 12H), 2.97 (br s, 12H), 2.74 (br s, 24H), 2.61 (d, *J* = 12.7 Hz, 12H). ^13^C{^1^H} NMR (126 MHz, DMSO-*d*_6_): δ = 141.64, 139.94, 139.78, 127.78, 127.32, 125.14, 53.46, 51.98, 48.26. ^19^F NMR (470 MHz, DMSO-*d*_6_): δ = −78.0 (s) ppm. Calculated elemental analysis for C_136_H_144_N_16_O_12_F_12_S_4_Ag_4_ (**2-Ag**): C, 54.77%; H, 4.87%; N, 7.51%. Found: C, 53.81%; H, 5.07%; N, 7.61%.

## Supporting Information Available

Nuclear magnetic resonance data for all newly reported compounds; additional gas sorption data and information; information on isotherm data fitting; details of crystallographic data collection and refinement; additional images showing the solid-state structures of newly reported compounds; thermogravimetric analysis data for all newly reported compounds (PDF)
