# Supplementary material for: Installation of Copper(I) and Silver(I) Sites into TREN-Based Porous Organic Cages via Postsynthetic Metalation
Source: Organometallics. 2024 Sep 12;43(20):2599–607. doi: 10.1021/acs.organomet.4c00247 (PMC11523223; doi:10.1021/acs.organomet.4c00247)
Supplement: Supplementary file 1 — om4c00247_si_001.pdf [file om4c00247_si_001.pdf]

Supporting Information for the Paper Entitled:

Installation of Copper(I) and Silver(I) Sites into TREN-based Porous Organic Cages via Post-Synthetic Metallation

Hope A. Silva[a], Bevan S. Whitehead[a], Christopher D. Hastings[a], Chandan Kumar Tiwari[a], William W. Brennessel[a], and Brandon R. Barnett[a]*

[a]*Department of Chemistry, University of Rochester, Rochester, NY, USA*

**Email: brandon.barnett@rochester.edu*

Table of Contents

1. Details of Gas Sorption Isotherm Measurements…S2

2. Infrared Spectroscopic Measurements………..S13

3. Electronic Spectroscopy Measurements…………S15

4. Details of Crystallographic Structure Determinations…S16

5. Nuclear Magnetic Resonance Spectroscopic Data…..S25

6. Thermogravimetric Analysis Data……S33

7. References……………S37

1. Details of Gas Sorption Isotherm Measurements.

Adsorption isotherm measurements were performed on a Micromeritics 3Flex physisorption analyzer. Gas purities for all adsorbates were of at least 99.99% purity. For macrocycles **1** and **2**, an equilibration interval of 100 s was employed for all isotherms except for CO_2 (180 s). For macrocycle **1-Cu**, an equilibration interval of 180 s was employed for all adsorbates except methane (CH_4) and ethylene (C_2H_4), for which 90 s equilibration intervals were implemented. For **2-Cu** and **1-Ag**, an equilibration interval of 180 s was used for all adsorbates.

Isotherm Fitting, Differential Enthalpies of Adsorption, and IAST Calculations. Single-component adsorption isotherms were fit individually using a single-site Langmuir Freundlich equation, given by:

$$n = \frac{q_{(sat)} b\, p^{v}}{1 + b\, p^{v}} \tag{1}$$

where n is the amount adsorbed in mmol/g, q_{sat} is the amount adsorbed when saturated with the gas in mmol/g, b is the Langmuir parameter in bar^{-1}, P is the gas pressure in bar, and v is the dimensionless Freundlich parameter. These values were determined using a least-squares method and are given in Tables S1-S4.

Ideal Adsorbed Solution Theory (IAST) was used to determine adsorbent selectivity from single-component gas adsorption isotherms.[1] This approach involves numerically solving for the spreading pressure and subsequently determining the composition of the adsorbed phase at a given gas phase composition. The selectivity S for adsorption of a from a two-component mixture comprised of a and b is then given by:

$$S = \frac{x_a / x_b}{y_a / y_b} \tag{2}$$

where x is the mole fraction in the adsorbed phase, y is the mole fraction in the gas phase, and the subscripts a and b refer to the mixture components.

Brunauer-Emmett-Teller (BET) Surface Area Analyses.

The general form of the BET equation is,

$$\frac{x}{v(1-x)} = \frac{1}{v_m c} + \frac{(c-1)}{v_m c} \cdot x \tag{3}$$

where v is the adsorption value at the relative pressure, $x = P/P_0$, which is the equilibrium pressure normalized by the saturation vapor pressure (P_0) of the adsorbate at the same temperature, v_m is the adsorption value corresponding to the monolayer, and $c = \exp(E_1\text{-}E_L)/RT$.

$$v(P_0 - P) = \frac{P_0 V_m c x}{1 + x(c+1)} \tag{4}$$

If we differentiate equation (4) with respect to x, we obtain,

$$\frac{d\big(v(P_0 - P)\big)}{dx} = \frac{d}{dx}\left(\frac{P_0 v_m c x}{1 + x(c+1)}\right) \tag{5}$$

$$= \frac{P_0 v_m c}{\left(1 + x(c-1)\right)^2}$$

Here, P_0 and v_m are positive values, and c should be a positive value to satisfy the second consistency criterion.

Using the 273 K carbon dioxide isothermal data chosen to satisfy the consistency criteria,[2] a linear fit can be calculated with the equation y=mx+b. This fit can then be used to calculate the c and v_m values using equation (5). These values could then be plugged into equation (6):

$$BET\ Surface\ Area = v_m \sigma_0 N_{av} \ (6)$$

Where σ_0 is equal to the cross-sectional area of CO_2 at liquid density (14.2 Å) and N_{av} is Avogadro's number.

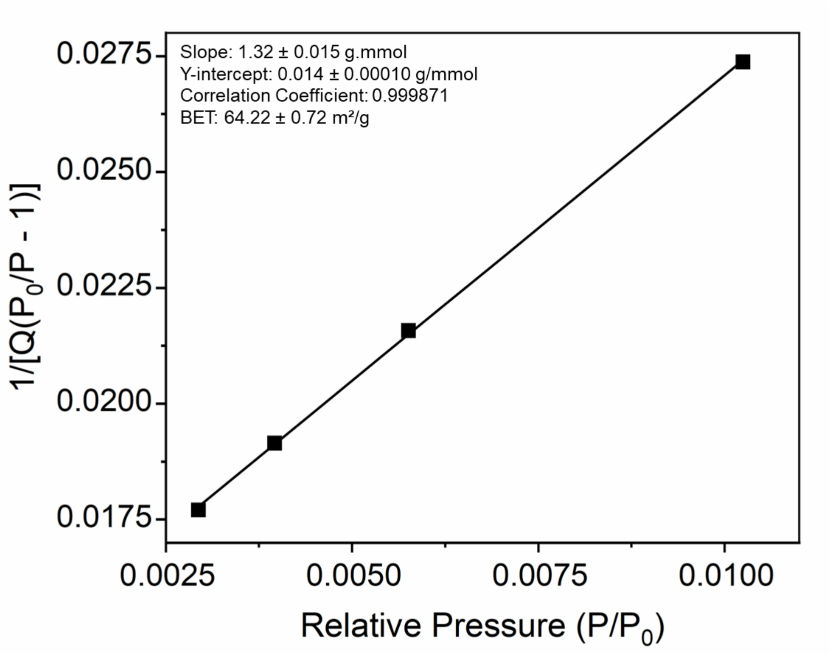

Figure S1. BET fitting of **1** within the range of 0.0025 < x < 0.012. Using the BET equation, c = 96 and v_m = 0.75 mmol/g were calculated. Using equation (6), Area=$v_m\sigma_0N_{av}$, a BET surface area of 64.22 ± 0.72 m²/g was calculated.

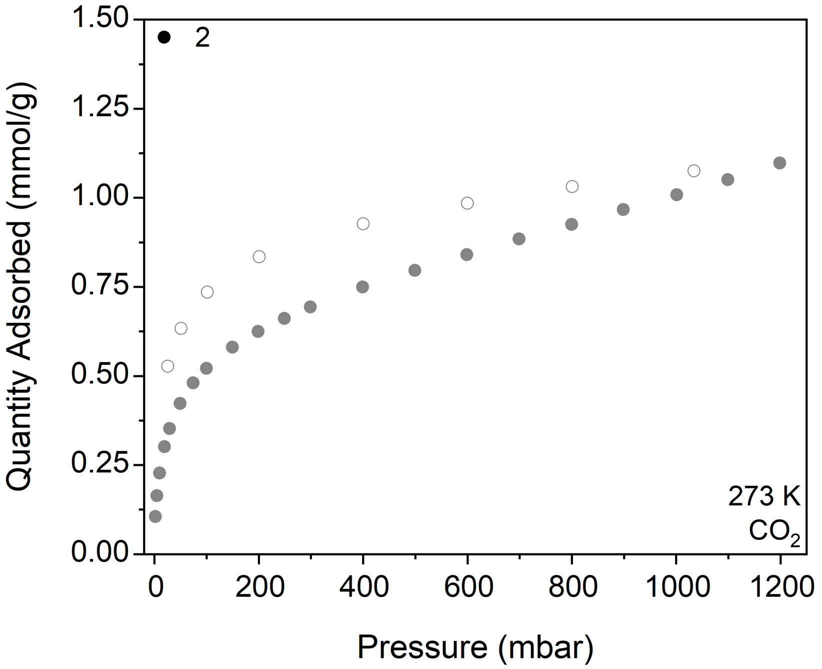

Figure S2. Carbon dioxide isotherm of **2** collected at 273 K.

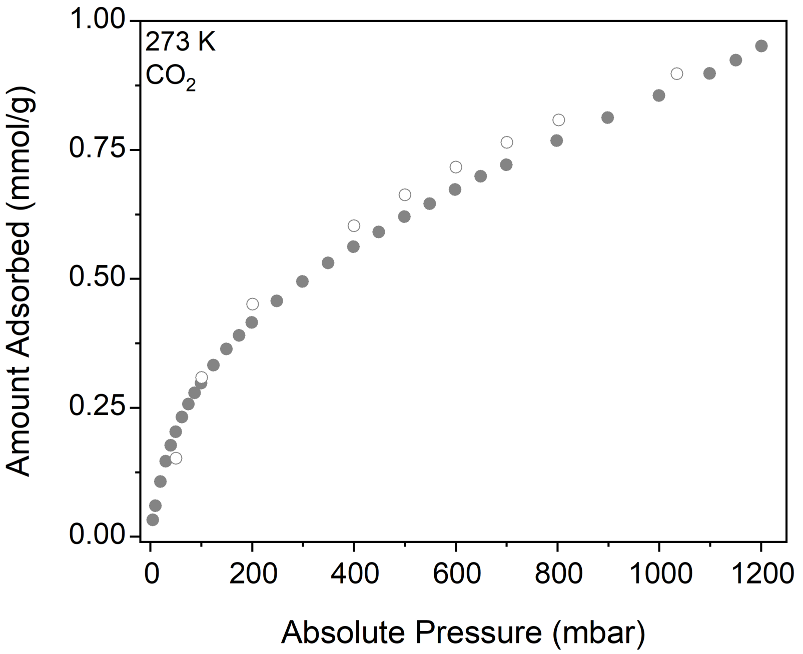

Figure S3. Carbon dioxide isotherm of **1-Cu** collected at 273 K.

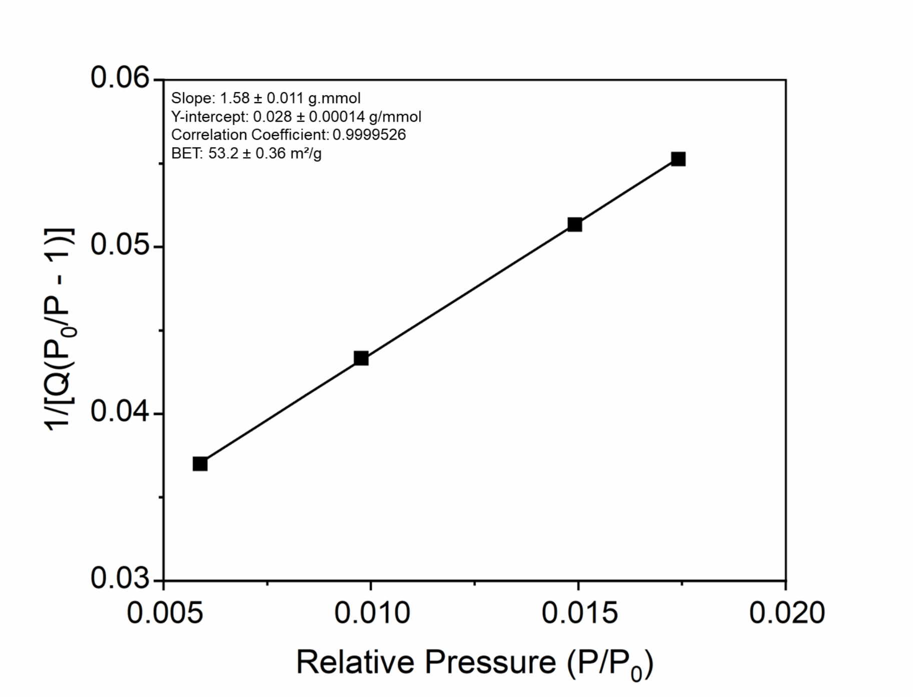

Figure S4. BET fitting of **1-Cu** within the range of 0.005 < x < 0.018. Using the BET equation, c = 58 and v_m = 0.62 mmol/g were calculated. Using equation (6), Area=$v_m\sigma_0N_{av}$, a BET surface area of 53.2 ± 0.36 m²/g was calculated.

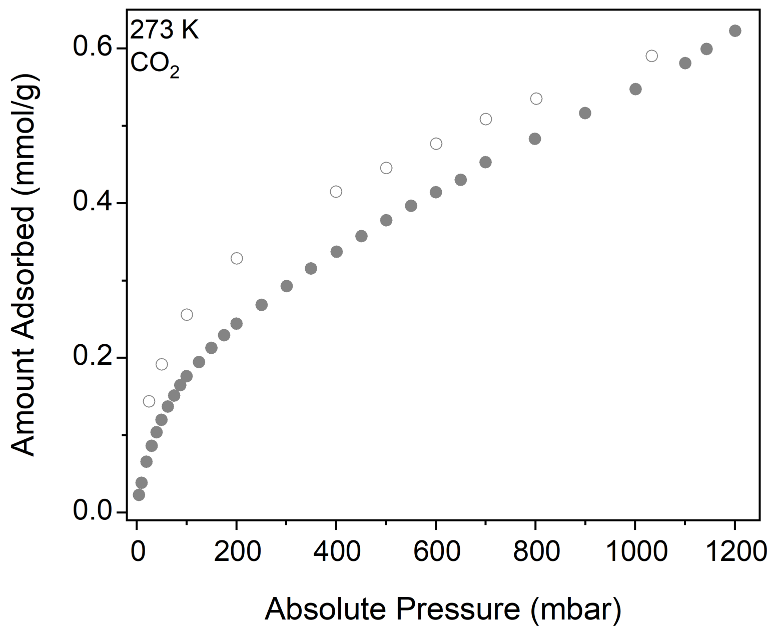

Figure S5. Carbon dioxide isotherm of **2-Cu** collected at 273 K.

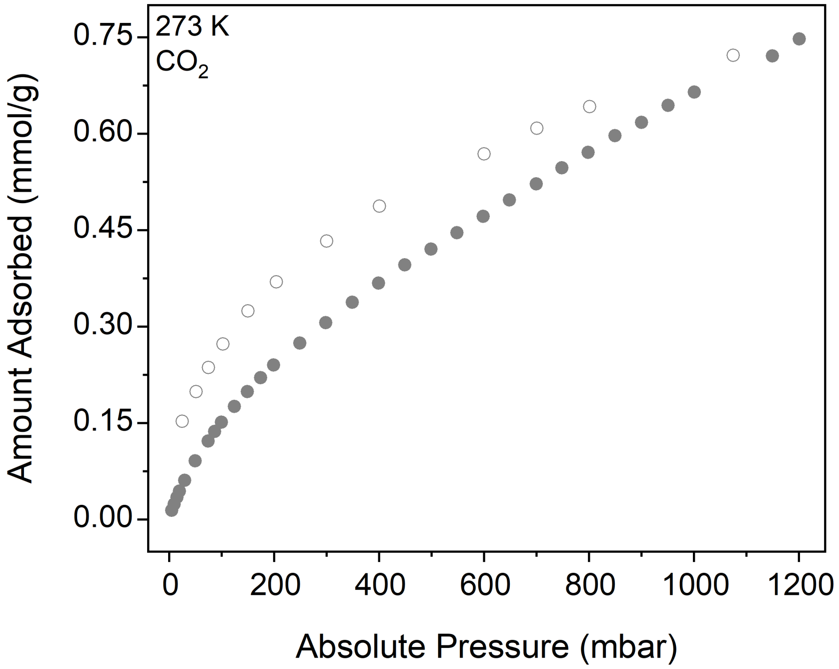

Figure S6. Carbon dioxide isotherm of **1-Ag** collected at 273 K.

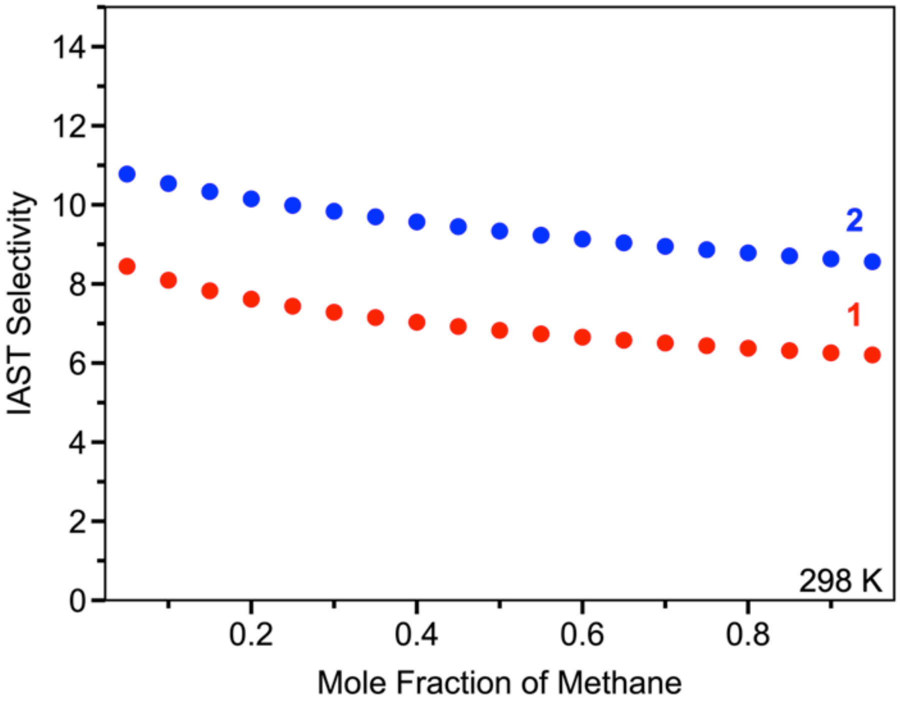

Figure S7. Data obtained from IAST selectivity of **1** and **2** calculations for methane/nitrogen mixture. Calculations were performed assuming $T = 298$ K and $P_{tot} = 1$ bar.

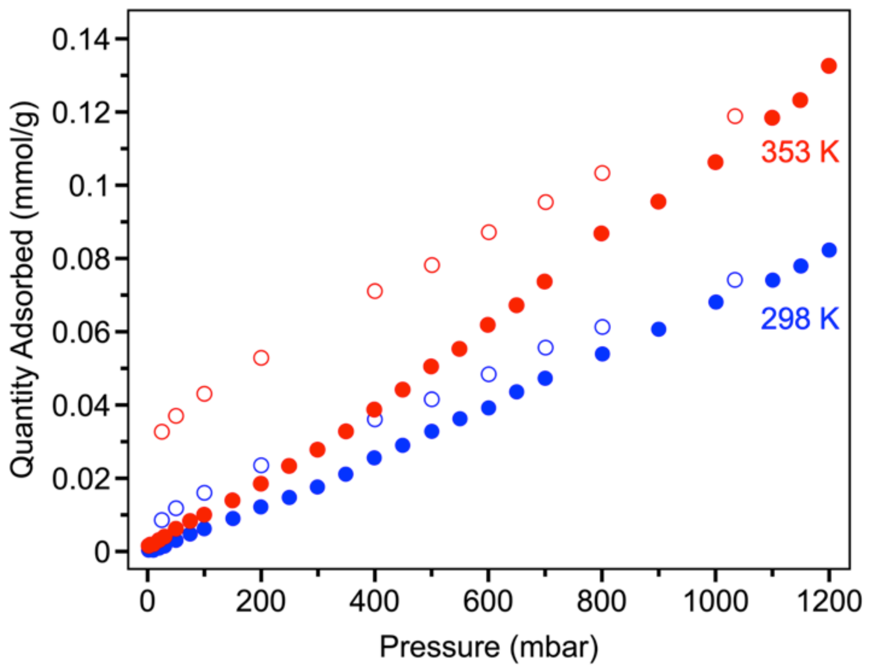

Figure S8. Oxygen adsorption data for **1-Cu** at 298 K and 353 K.

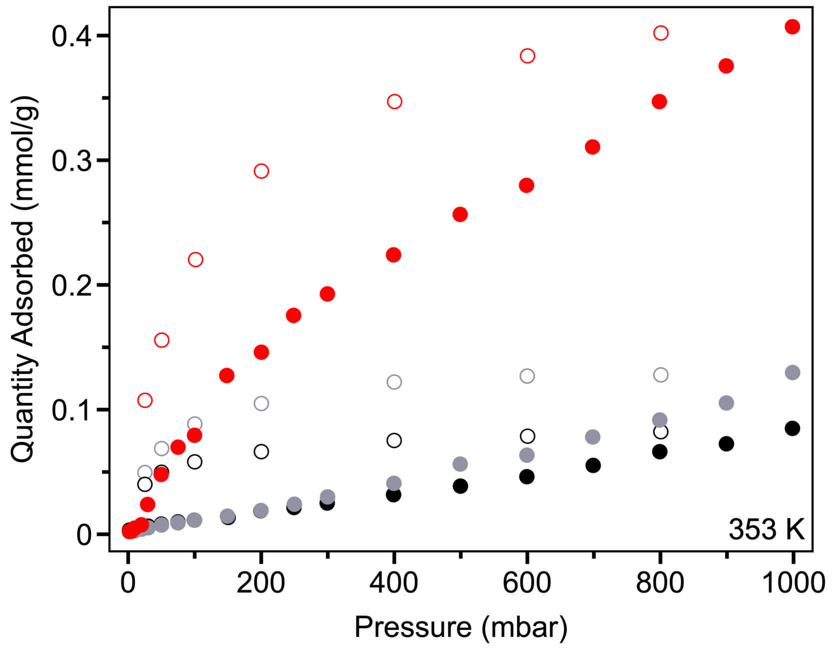

Figure S9. Oxygen adsorption data for **2-Cu** at 353 K. The red data points corresponded to the first isotherm collected. Following reactivation at 353 K, a second oxygen isotherm was collected (grey data points). Following reactivation at 423 K, a third oxygen isotherm was collected (black data points).

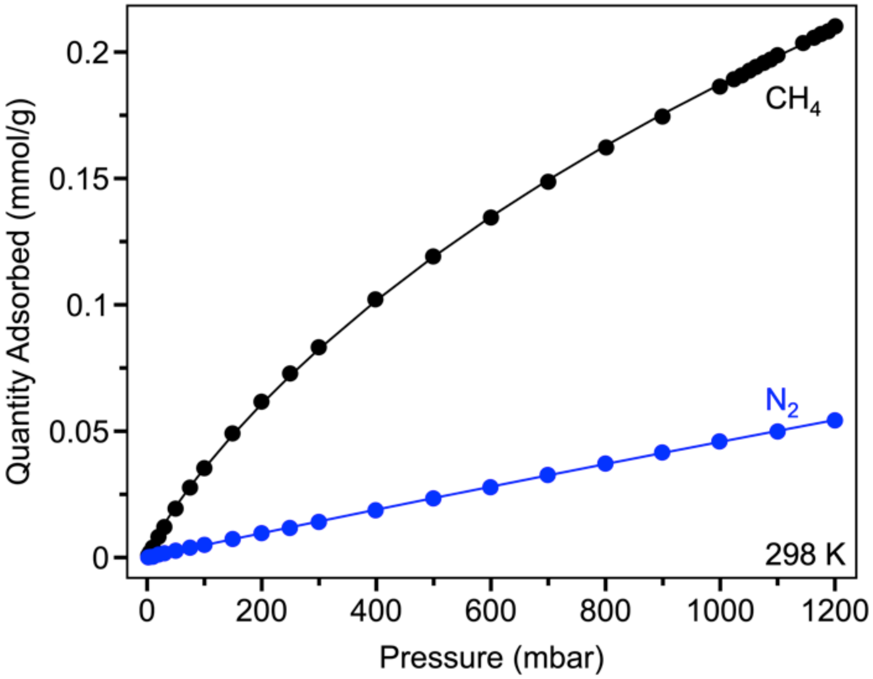

Figure S10. CH_4 and N_2 adsorption isotherms (data points) and corresponding Langmuir-Freundlich fits for **1** (solid lines) at 298 K.

Table S1. Single-site Langmuir-Freundlich fit parameters for **1** for CH_4 and N_2.

**Gas**	**Methane**	**Nitrogen**
q_{sat} **(mmol/g)**	0.68	0.52
b **(bar^{-1})**	0.38	0.097
v	0.84	1.02

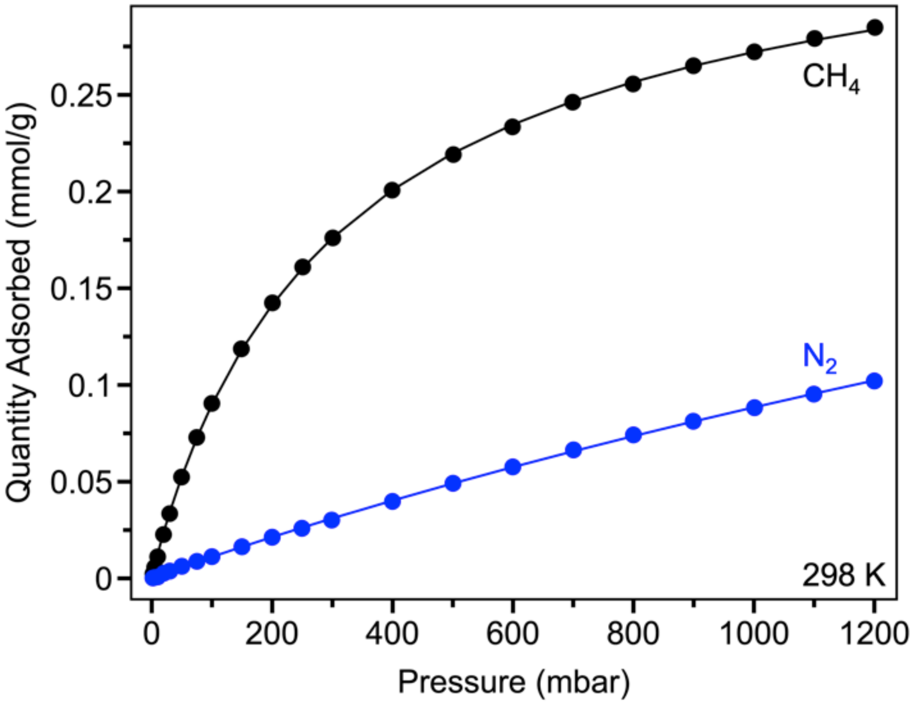

Figure S11. CH_4 and N_2 adsorption isotherms (data points) and corresponding Langmuir-Freundlich fits for **2** (solid lines) at 298 K.

Table S2. Single-site Langmuir-Freundlich fit parameters for **2** for CH_4 and N_2.

Gas	Methane	Nitrogen
q_{sat} **(mmol/g)**	0.37	0.51
b **(bar^{-1})**	2.93	0.210
v	0.95	0.98

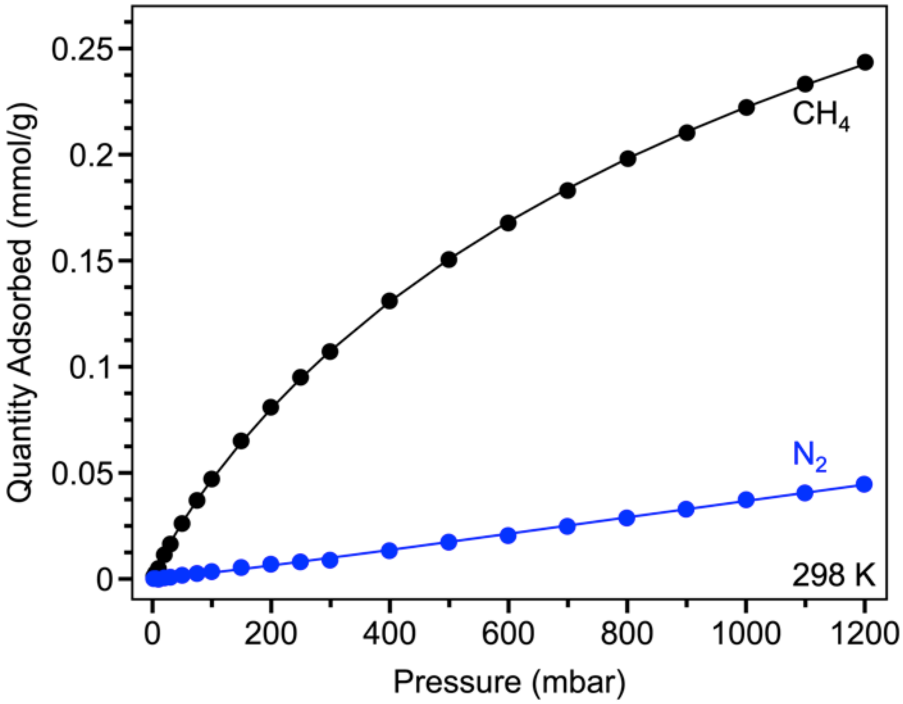

Figure S12. CH_4 and N_2 adsorption isotherms (data points) and corresponding Langmuir-Freundlich fits for **1-Cu** (solid lines) at 298 K.

Table S3. Single-site Langmuir-Freundlich fit parameters for **1-Cu** for CH_4 and N_2.

Gas	Methane	Nitrogen
q_{sat} (mmol/g)	0.50	0.52
b (bar^{-1})	0.79	0.08
v	0.89	1.13

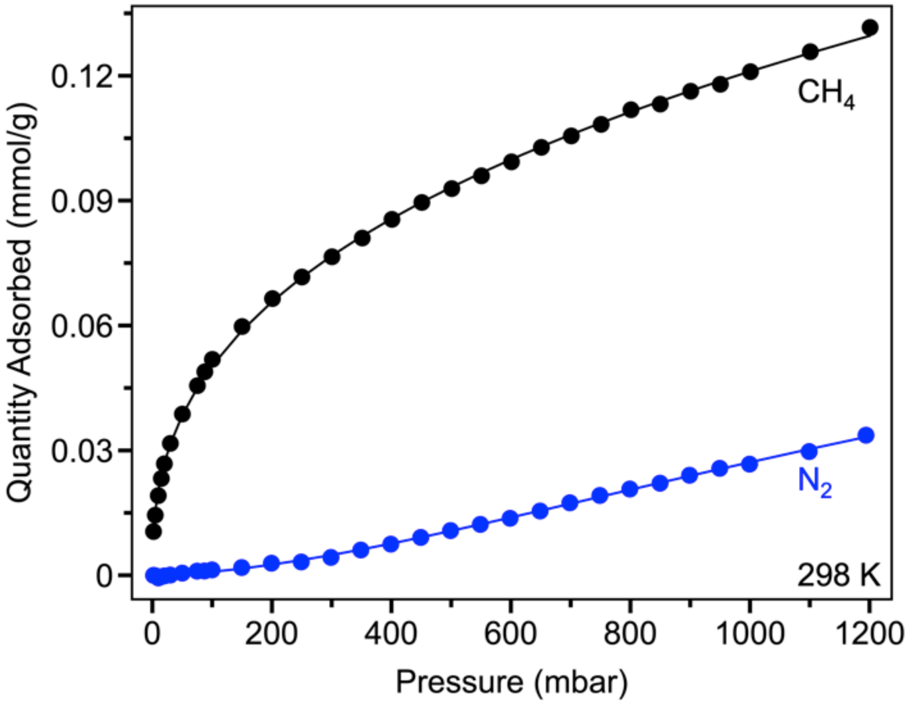

Figure S13. CH_4 and N_2 adsorption isotherms (data points) and corresponding Langmuir-Freundlich fits for **2-Cu** (solid lines) at 298 K.

Table S4. Single-site Langmuir-Freundlich fit parameters for **2-Cu** for CH_4 and N_2.

Gas	**Methane**	**Nitrogen**
q_{sat} **(mmol/g)**	2.28	0.10
b **(bar^{-1})**	0.06	0.40
v	0.40	1.66

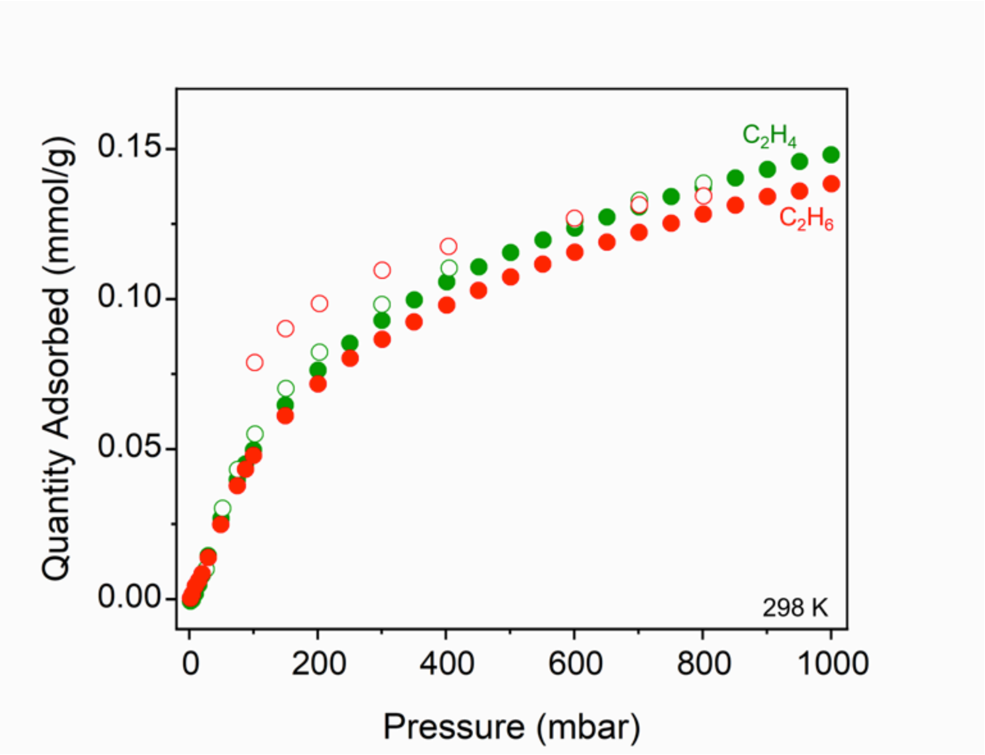

Figure S14. Ethylene and ethane sorption isotherms for **1-Ag** at 298 K. Filled and open circles correspond to data obtained during adsorption and desorption sweeps, respectively.

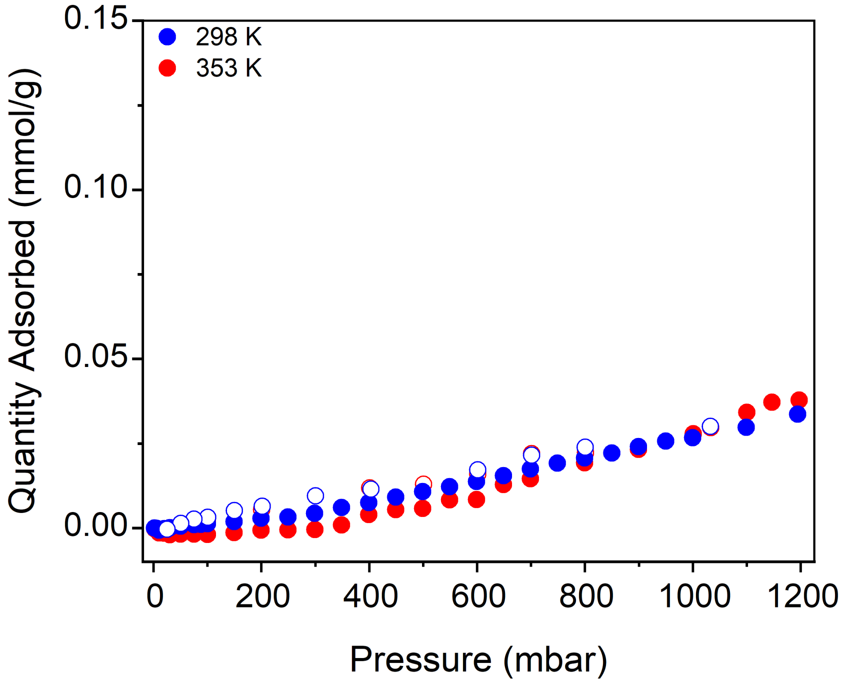

Figure S15. Nitrogen sorption isotherms for **2-Cu** at 298 K and 353 K. Filled and open circles correspond to data obtained during adsorption and desorption sweeps, respectively.

2. Infrared Spectroscopy

Attenuated total reflectance (ATR) or transmission (FT-IR) infrared spectra of the organic cages were collected on a Perkin-Elmer Spectrum 3 spectrometer housed within a nitrogen-filled glovebox. FT-IR samples were prepared as a pressed pellet diluted in anhydrous potassium bromide (KBr).

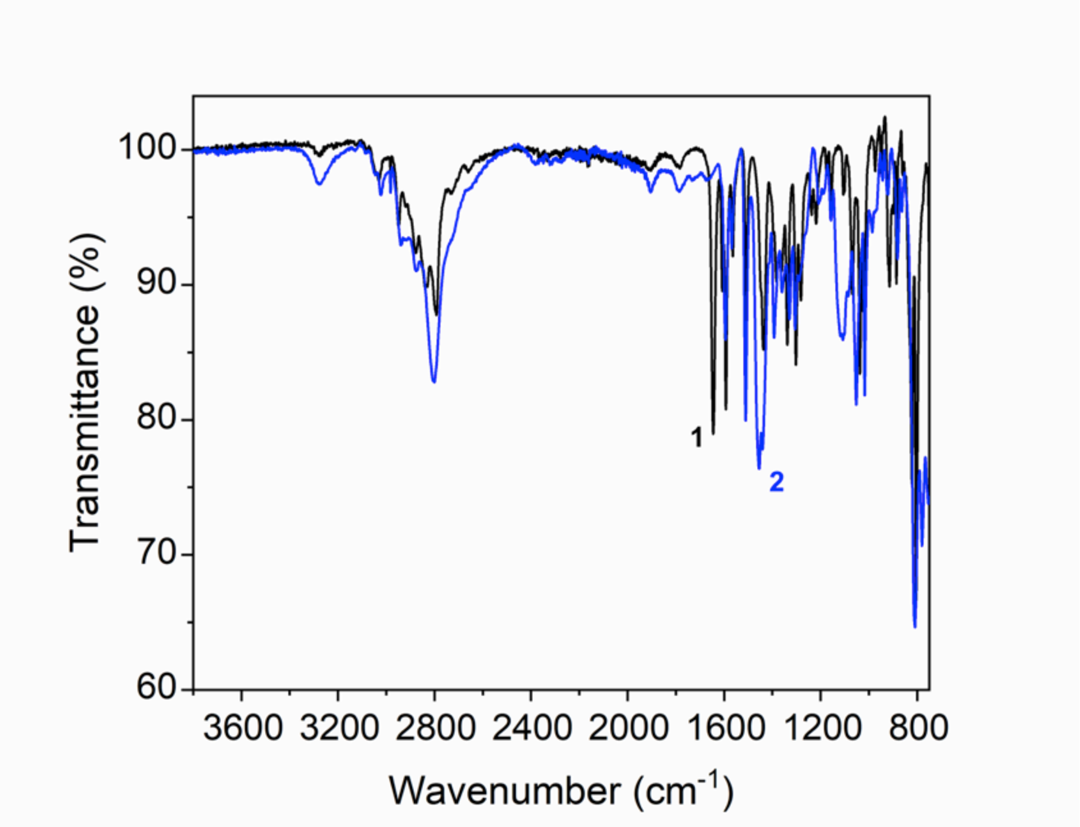

Figure S16. Comparative ATR-IR spectra of **1** (black) and **2** (blue).

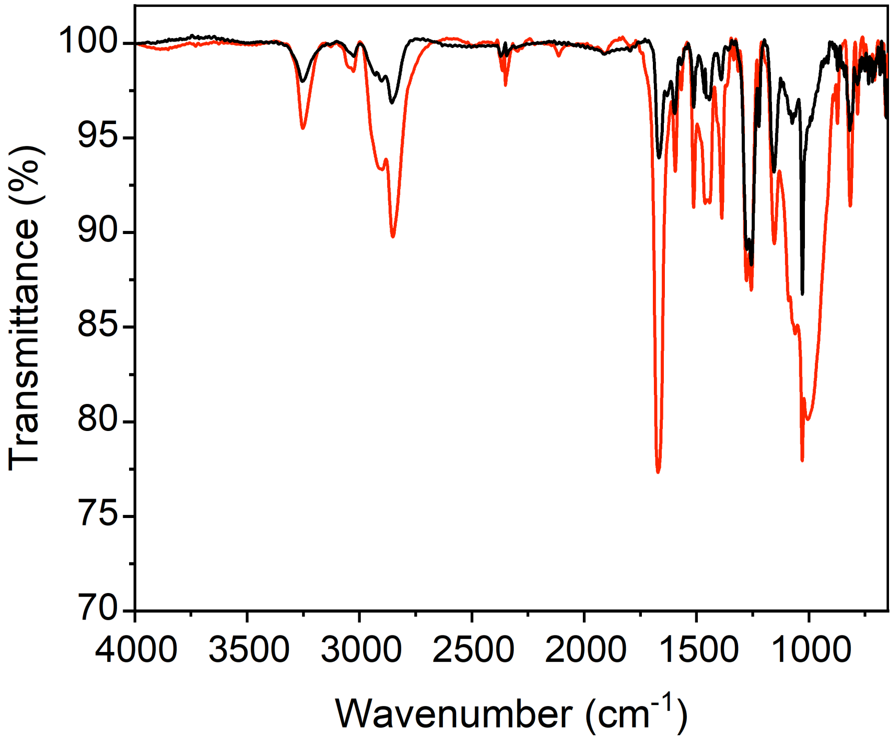

Figure S17. Comparative FT-IR spectra of **2-Cu** before (red) and after (black) exposure to 1 bar O_2 at 353 K.

3. Electronic Spectroscopy

Electronic spectra were collected on an Agilent Cary 60 spectrometer at ambient temperature. Samples were prepared in a quartz cuvette as DMF solutions.

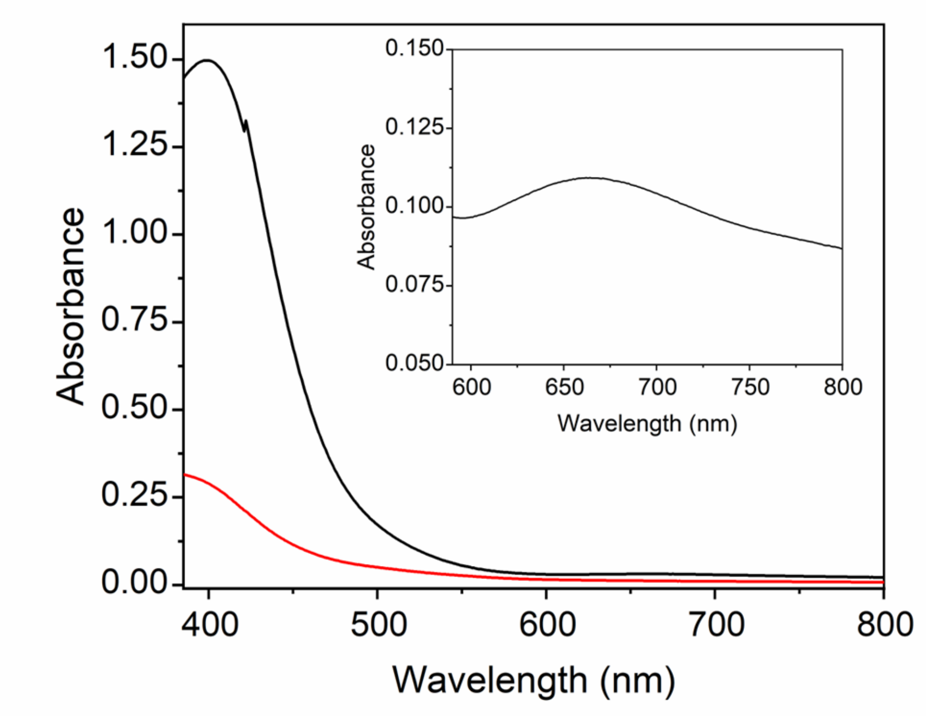

Figure S18. Electronic spectra of pristine **2-Cu** (red) and of **2-Cu** following exposure to 1 atm O_2 at 353 K (black). The inset depicts the weak band at higher wavelengths that appears following high temperature O_2 exposure.

4. Details of Crystallographic Structure Determinations

Details of data collection, structure solution, and refinement.

2•$CHCl_3$. A crystal (0.248 x 0.165 x 0.092 mm^3) was placed onto a nylon loop and mounted on a Rigaku XtaLAB Synergy-S Dualflex diffractometer equipped with a HyPix-6000HE HPC area detector for data collection at 100.00(10) K. A preliminary set of cell constants and an orientation matrix were calculated from a small sampling of reflections.[3] A short pre-experiment was run, from which an optimal data collection strategy was determined. The full data collection was carried out using a PhotonJet (Cu) X-ray source with frame times of 11.00 and 33.00 seconds and a detector distance of 34.0 mm. Series of frames were collected in 0.50º steps in *w* at different 2*q*, *k*, and *f* settings. After the intensity data were corrected for absorption, the final cell constants were calculated from the xyz centroids of 39827 strong reflections from the actual data collection after integration.[3] The higher angle intensity data were weak or non-existent, likely due to severe disorder and/or solvent loss. See Table S5 for additional crystal and refinement information.

The structure was solved using SHELXT[4] and refined using SHELXL.[5] The space group $P2_1/n$ was determined based on systematic absences. Most or all non-hydrogen atoms were assigned from the solution. Full-matrix least squares / difference Fourier cycles were performed which located any remaining non-hydrogen atoms. All non-hydrogen atoms were refined with anisotropic displacement parameters. All hydrogen atoms were placed in ideal positions and refined as riding atoms with relative isotropic displacement parameters.

Reflection contributions from multiple chloroform solvent molecules (some ordered, most disordered, approx. 20-22 in total, 10-11 per N_{16} molecule) were fixed and added to the calculated structure factors using the SQUEEZE routine of program Platon,[6] which determined there to be 4923 electrons in 11449 Å^3 per unit cell treated this way. This solvent was not included in the atom list or molecular formula (the caged chloroform molecules were modeled), and thus all calculated quantities that derive from the molecular formula (e.g., F(000), density, molecular weight, etc.) are known to be inaccurate. The final full matrix least squares refinement converged to $R1 = 0.2058$ (F^2, $I > 2s(I)$) and $wR2 = 0.5836$ (F^2, all data).

1-Cu. A crystal (0.273 x 0.223 x 0.152 mm^3) was placed onto a thin glass optical fiber or a nylon loop and mounted on a Rigaku XtaLAB Synergy-S Dualflex diffractometer equipped with a HyPix-6000HE HPC area detector for data collection at 100.00(10) K. A preliminary set of cell constants and an orientation matrix were calculated from a small sampling of reflections.[3] A short pre-experiment was run, from which an optimal data collection strategy was determined. The full data collection was carried out using a PhotonJet (Cu) X-ray source with frame times of 8.73 and 54.00 seconds and a detector distance of 34.0 mm. Series of frames were collected in 0.50º steps in *w* at different 2*q*, *k*, and *f* settings. After the intensity data were corrected for absorption, the final cell constants were calculated from the xyz centroids of 3507 strong reflections from the actual data collection after integration.[3] See Table S6 for additional crystal and refinement information.

The structure was solved using SHELXT[4] and refined using SHELXL.[5] The space group *P*-43*n* was determined based on systematic absences and intensity statistics. Most or all non-hydrogen atoms were assigned from the solution. Full-matrix least squares / difference Fourier

cycles were performed which located any remaining non-hydrogen atoms. All non-hydrogen atoms were refined with anisotropic displacement parameters. All hydrogen atoms were placed in ideal positions and refined as riding atoms with relative isotropic displacement parameters.

Reflection contributions from highly disordered solvent and anions were fixed and added to the calculated structure factors using the SQUEEZE routine of program Platon,[6] which determined there to be 2082 electrons in 6274 $Å^3$ per unit cell treated this way. Because of the nature of this cage structure, there are small void volumes in the center of the cages (30 electrons in 164 $Å^3$) and large volumes between cages (2021 electrons in 5938 $Å^3$). The triflate anions, four per Cu_4 cage, are known to exist from the synthesis and independent spectroscopic analyses. These anions were highly disordered and could not be modeled appropriately. However, it is important to note that one triflate anion consists of 74 electrons, which is considerably larger than the number of electrons found in the intracage void, corroborating the notion that all triflates reside outside of the cage. Because the exact identity and amount of the atoms in the void spaces were unknown, they were not included in the atom list or molecular formula. Thus all calculated quantities that derive from the molecular formula are known to be inaccurate. The final full matrix least squares refinement converged to $R1 = 0.0396$ (F^2, $I > 2s(I)$) and $wR2 = 0.1490$ (F^2, all data).

2-Cu. A crystal (0.36 x 0.13 x 0.03 mm^3) was placed onto a nylon loop and mounted on a Rigaku XtaLAB Synergy-S Dualflex diffractometer equipped with a HyPix-6000HE HPC area detector for data collection at 100.01(10) K. A preliminary set of cell constants and an orientation matrix were calculated from a small sampling of reflections.[3] A short pre-experiment was run, from which an optimal data collection strategy was determined. The full data collection was carried out using a PhotonJet (Cu) X-ray source with frame times of 16.00 and 80.00 seconds and a detector distance of 34.0 mm. Series of frames were collected in 0.50º steps in w at different $2q$, k, and f settings. After the intensity data were corrected for absorption, the final cell constants were calculated from the xyz centroids of 26552 strong reflections from the actual data collection after integration.[3] See Table S7 for additional crystal and refinement information.

The structure was solved using SHELXT[4] and refined using SHELXL.[5] The space group *Pbca* was determined based on systematic absences. Most or all non-hydrogen atoms were assigned from the solution. Full-matrix least squares / difference Fourier cycles were performed which located any remaining non-hydrogen atoms. All non-hydrogen atoms were refined with anisotropic displacement parameters. The N-H hydrogen atoms were found from the difference Fourier map and then given riding models. All other hydrogen atoms were placed in ideal positions and refined as riding atoms with relative isotropic displacement parameters.

Reflection contributions from highly disordered solvent were fixed and added to the calculated structure factors using the SQUEEZE routine of program Platon,[6] which determined there to be 5180 electrons in 14046 $Å^3$ per unit cell treated this way. Because the exact identity and amount of the disordered species in the voids were not known, they were not included in the atom list or molecular formula. Thus all calculated quantities that derive from the molecular formula (e.g., F(000), density, molecular weight, etc.) are known to be inaccurate. The final full matrix least squares refinement converged to $R1 = 0.0837$ (F^2, $I > 2s(I)$) and $wR2 = 0.2739$ (F^2, all data).

1-Ag. A crystal (0.151 x 0.14 x 0.127 mm^3) was placed onto a thin glass optical fiber or a nylon loop and mounted on a Rigaku XtaLAB Synergy-S Dualflex diffractometer equipped with a HyPix-6000HE HPC area detector for data collection at 100.00(10) K. A preliminary set of cell constants and an orientation matrix were calculated from a small sampling of reflections.[3] A short pre-experiment was run, from which an optimal data collection strategy was determined. The full data collection was carried out using a PhotonJet (Cu) X-ray source with frame times of 6.58 and 40.00 seconds and a detector distance of 34.0 mm. Series of frames were collected in 0.50º steps in *w* at different 2*q*, *k*, and *f* settings. After the intensity data were corrected for absorption, the final cell constants were calculated from the xyz centroids of 8702 strong reflections from the actual data collection after integration.[3] See Table S8 for additional crystal and refinement information.

The structure was solved using SHELXT[4] and refined using SHELXL.[5] The space group *P*-43*n* was determined based on systematic absences and intensity statistics. Most or all non-hydrogen atoms were assigned from the solution. Full-matrix least squares / difference Fourier cycles were performed which located any remaining non-hydrogen atoms. All non-hydrogen atoms were refined with anisotropic displacement parameters. All hydrogen atoms were placed in ideal positions and refined as riding atoms with relative isotropic displacement parameters.

Reflection contributions from highly disordered solvent and anions were fixed and added to the calculated structure factors using the SQUEEZE routine of program Platon,[6] which determined there to be 2268 electrons in 7424 Å^3 per unit cell treated this way. Note that, unlike **1-Cu**, Platon did not differentiate between the intrinsic and extrinsic voids. The triflate anions, four per Ag_4 cage, are known to exist from the synthesis and independent spectroscopic analyses. These anions were highly disordered and could not be modeled appropriately. However, owing to the structural similarities between these compounds and the porosity observed from gas sorption isotherms, we can assume with high confidence that the triflate anions reside outside of the cages. Because the exact identity and amount of the atoms in the void space were unknown, they were not included in the atom list or molecular formula. Thus all calculated quantities that derive from the molecular formula are known to be inaccurate. The final full matrix least squares refinement converged to $R1 = 0.0365$ (F^2, $I > 2s(I)$) and $wR2 = 0.1082$ (F^2, all data).

Table S5. Crystal data and structure refinement information for **2**•$CHCl_3$.

Identification code	brbhas01	
Empirical formula	C133 H145 Cl3 N16	
Formula weight	2073.99	
Temperature	100.00(10) K	
Wavelength	1.54184 Å	
Crystal system	monoclinic	
Space group	$P2_1/n$	
Unit cell dimensions	*a* = 21.7379(2) Å	a = 90°
	b = 42.7386(5) Å	b = 101.5210(10)°
	c = 34.5465(4) Å	g = 90°
Volume	31448.7(6) $Å^3$	
Z	8	
Density (calculated)	0.876 Mg/m^3	
Absorption coefficient	0.853 mm^{-1}	
F(000)	8848	
Crystal color, morphology	colourless, block	
Crystal size	0.248 x 0.165 x 0.092 mm^3	
Theta range for data collection	2.318 to 80.887°	
Index ranges	-16 £ *h* £ 26, -44 £ *k* £ 40, -44 £ *l* £ 41	
Reflections collected	117899	
Independent reflections	42263 [*R*(int) = 0.0616]	
Observed reflections	25122	
Completeness to theta = 67.684°	72.2%	
Absorption correction	Multi-scan	
Max. and min. transmission	1.00000 and 0.24265	
Refinement method	Full-matrix least-squares on F^2	
Data / restraints / parameters	42263 / 2858 / 2904	
Goodness-of-fit on F^2	2.330	
Final *R* indices [*I*>2sigma(*I*)]	*R*1 = 0.2058, *wR*2 = 0.5586	
R indices (all data)	*R*1 = 0.2480, *wR*2 = 0.5836	
Largest diff. peak and hole	1.458 and -0.641 e.$Å^{-3}$	

Table S6. Crystal data and structure refinement for **1-Cu**.

Identification code	brbbrb06	
Empirical formula	C132 H120 Cu4 N16	
Formula weight	2184.59	
Temperature	100.00(10) K	
Wavelength	1.54184 Å	
Crystal system	cubic	
Space group	*P*-43*n*	
Unit cell dimensions	*a* = 21.9964(7) Å	a = 90°
	b = 21.9964(7) Å	b = 90°
	c = 21.9964(7) Å	g = 90°
Volume	10642.8(10) $Å^3$	
Z	2	
Density (calculated)	0.682 Mg/m^3	
Absorption coefficient	0.688 mm^{-1}	
F(000)	2280	
Crystal color, morphology	yellow, block	
Crystal size	0.273 x 0.223 x 0.152 mm^3	
Theta range for data collection	2.841 to 80.090°	
Index ranges	-20 £ *h* £ 17, -25 £ *k* £ 27, -25 £ *l* £ 19	
Reflections collected	15479	
Independent reflections	3648 [*R*(int) = 0.0341]	
Observed reflections	1948	
Completeness to theta = 67.684°	99.9%	
Absorption correction	Multi-scan	
Max. and min. transmission	1.00000 and 0.39417	
Refinement method	Full-matrix least-squares on F^2	
Data / restraints / parameters	3648 / 0 / 116	
Goodness-of-fit on F^2	0.917	
Final *R* indices [*I*>2sigma(*I*)]	*R*1 = 0.0396, *wR*2 = 0.1250	
R indices (all data)	*R*1 = 0.0756, *wR*2 = 0.1490	
Absolute structure parameter	0.15(7)	
Largest diff. peak and hole	0.114 and -0.098 e.$Å^{-3}$	

Table S7. Crystal data and structure refinement for **2-Cu**.

Identification code	brbbrb13r	
Empirical formula	C139 H151 Cu4 F12 N17 O13 S4	
Formula weight	2878.16	
Temperature	100.01(10) K	
Wavelength	1.54184 Å	
Crystal system	orthorhombic	
Space group	*Pbca*	
Unit cell dimensions	*a* = 41.6810(7) Å	a = 90°
	b = 18.1702(2) Å	b = 90°
	c = 50.2441(5) Å	g = 90°
Volume	38052.5(9) $Å^3$	
Z	8	
Density (calculated)	1.005 Mg/m^3	
Absorption coefficient	1.413 mm^{-1}	
F(000)	11968	
Crystal color, morphology	colourless, plate	
Crystal size	0.36 x 0.13 x 0.03 mm^3	
Theta range for data collection	2.755 to 76.655°	
Index ranges	-50 £ *h* £ 49, -21 £ *k* £ 22, -23 £ *l* £ 62	
Reflections collected	155596	
Independent reflections	37828 [*R*(int) = 0.0910]	
Observed reflections	21444	
Completeness to theta = 67.684°	99.7%	
Absorption correction	Multi-scan	
Max. and min. transmission	1.00000 and 0.56412	
Refinement method	Full-matrix least-squares on F^2	
Data / restraints / parameters	37828 / 293 / 1792	
Goodness-of-fit on F^2	1.051	
Final *R* indices [*I*>2sigma(*I*)]	*R*1 = 0.0837, *wR*2 = 0.2372	
R indices (all data)	*R*1 = 0.1333, *wR*2 = 0.2739	
Largest diff. peak and hole	0.798 and -1.205 e.$Å^{-3}$	

Table S8. Crystal data and structure refinement for **1-Ag**.

Identification code	brbbrb07	
Empirical formula	C132 H120 Ag4 N16	
Formula weight	2361.91	
Temperature	100.00(10) K	
Wavelength	1.54184 Å	
Crystal system	cubic	
Space group	*P*-43*n*	
Unit cell dimensions	*a* = 22.7626(2) Å	a = 90°
	b = 22.7626(2) Å	b = 90°
	c = 22.7626(2) Å	g = 90°
Volume	11794.1(3) Å^3	
Z	2	
Density (calculated)	0.665 Mg/m^3	
Absorption coefficient	2.841 mm^{-1}	
F(000)	2424	
Crystal color, morphology	colourless, block	
Crystal size	0.151 x 0.14 x 0.127 mm^3	
Theta range for data collection	3.884 to 79.163°	
Index ranges	-18 £ *h* £ 28, -25 £ *k* £ 19, -26 £ *l* £ 23	
Reflections collected	22233	
Independent reflections	3787 [*R*(int) = 0.0358]	
Observed reflections	2932	
Completeness to theta = 74.504°	99.7%	
Absorption correction	Multi-scan	
Max. and min. transmission	1.00000 and 0.56171	
Refinement method	Full-matrix least-squares on F^2	
Data / restraints / parameters	3787 / 0 / 116	
Goodness-of-fit on F^2	1.017	
Final *R* indices [*I*>2sigma(*I*)]	*R*1 = 0.0365, *wR*2 = 0.1023	
R indices (all data)	*R*1 = 0.0466, *wR*2 = 0.1082	
Absolute structure parameter	0.074(14)	
Largest diff. peak and hole	0.284 and -0.553 e.Å^{-3}	

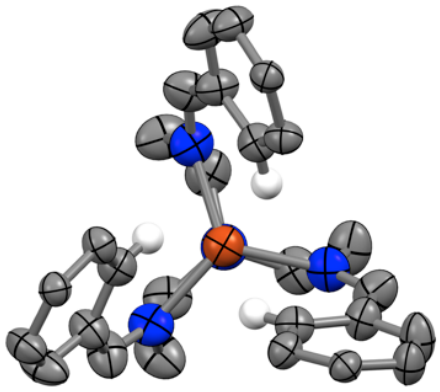

Figure S19. Close-up view of the Cu centers in **1-Cu**, looking down along the Cu–N_{axial} bond. An *ortho* C–H bond from each aryl ring sits in close proximity to the Cu site. Although the positions of these H-atoms were not refined, they lie approximately 2.43 Å from the metal center.

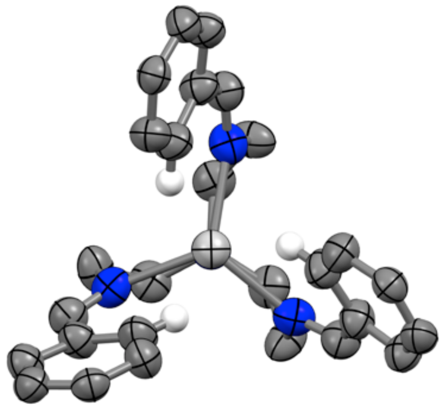

Figure S20. Close-up view of the Ag centers in **1-Ag**, looking down along the Ag–N_{axial} bond. An *ortho* C–H bond from each aryl ring sits in close proximity to the Ag site. Although the positions of these H-atoms were not refined, they lie approximately 2.42 Å from the metal center.

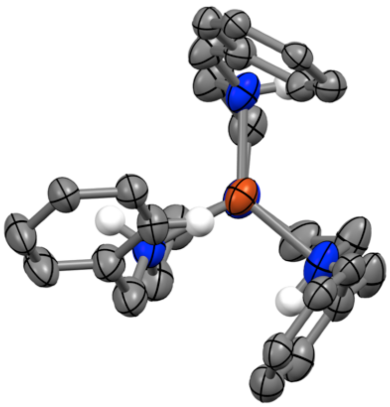

Figure S21. Close-up view of one of the Cu centers in **2-Cu**, looking down along the Cu–N_{axial} bond. Unlike in **1-Cu**, most Cu sites are not surrounded by three *ortho* C–H bonds from each aryl rings; for this particular Cu site, only one C–H bond points toward the metal, while the other two rings orient their faces inward.

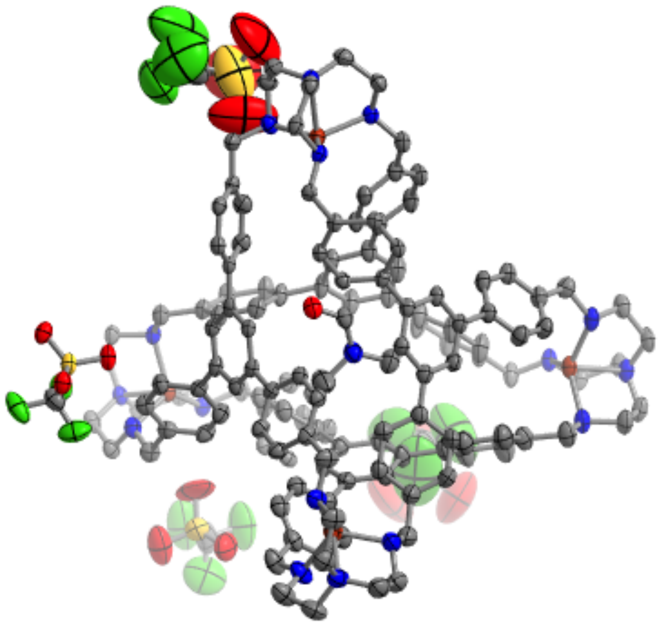

Figure S22. Solid-state structure of the tetracationic cage **2-Cu** showing the intracavity DMF guest molecule and the four triflate anions residing outside of the cavity.

Table S9. Calculated τ_4 values for **1-Cu**, **1-Ag**, and **1-Cu**.

Compound	Metal Center	Calculated τ_4 Value
**1-Cu**	Cu1[a]	0.86
**1-Ag**	Ag1[a]	0.91
**2-Cu**	Cu1	0.83
	Cu2	0.81
	Cu3	0.85
	Cu4	0.81

[a]All metal centers are crystallographically equivalent in these structures.

5. Nuclear Magnetic Resonance Spectra

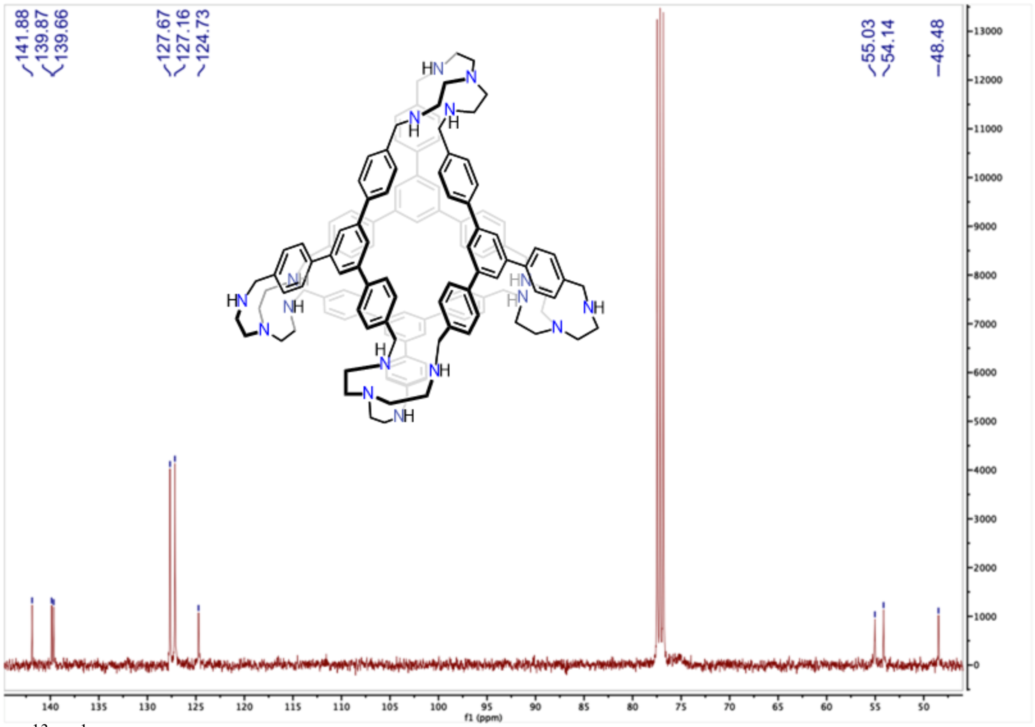

Figure S23. $^{13}C\{^{1}H\}$ NMR spectrum (101 MHz, 23 ºC) of **2** in $CDCl_3$. The unpicked triplet centered at 77.16 ppm corresponds to the $CDCl_3$ solvent residual.

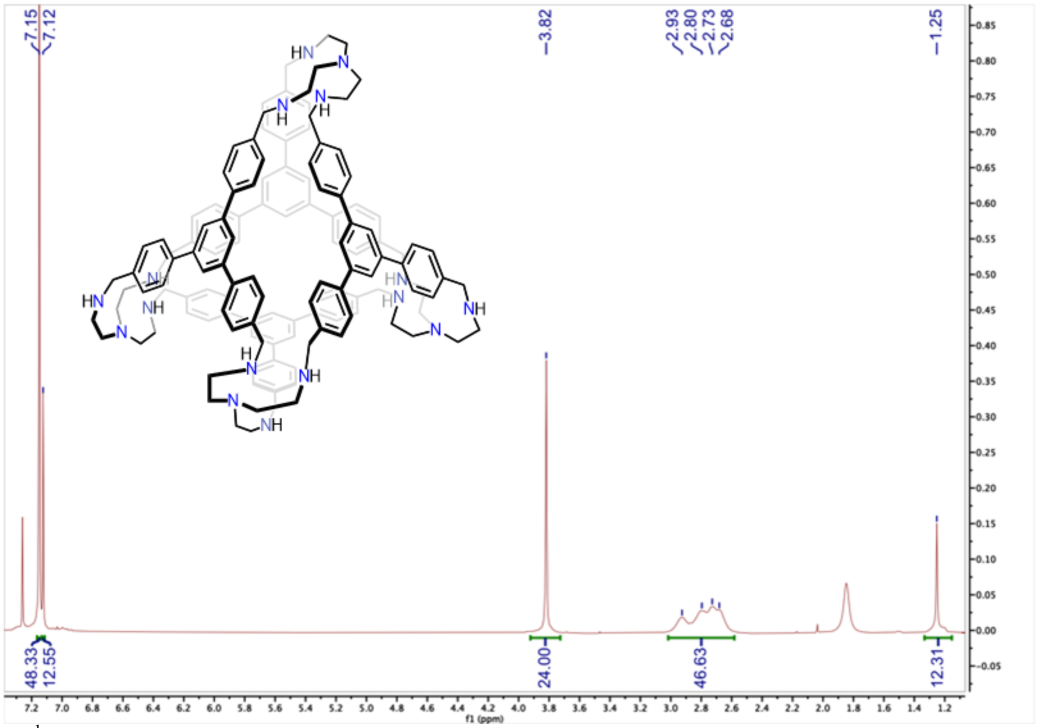

Figure S24. ^{1}H NMR (500 MHz, 23 ºC) spectrum of **2** in $CDCl_3$. The unpicked triplet at 7.26 ppm corresponds to the $CDCl_3$ solvent residual. The unpicked broad singlet at 1.83 corresponds to water.

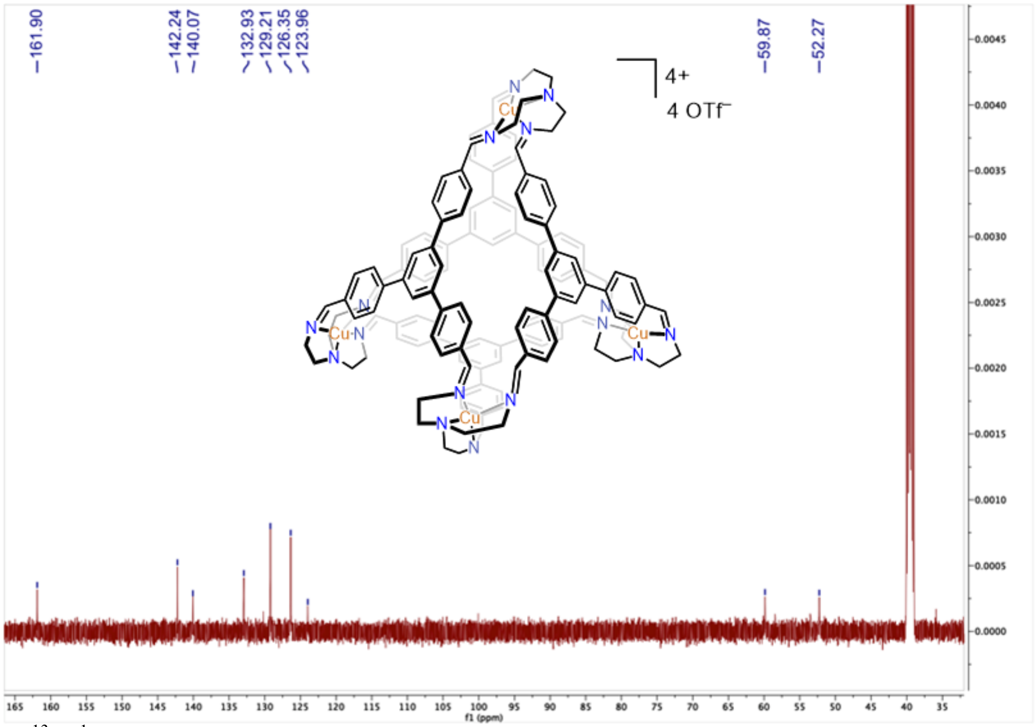

Figure S25. $^{13}C\{^{1}H\}$ NMR (126 MHz, 23 ºC) spectrum of **1-Cu** in DMSO-d_6. The unpicked multiplet centered at 39.52 ppm corresponds to the DMSO-d_6 solvent residual.

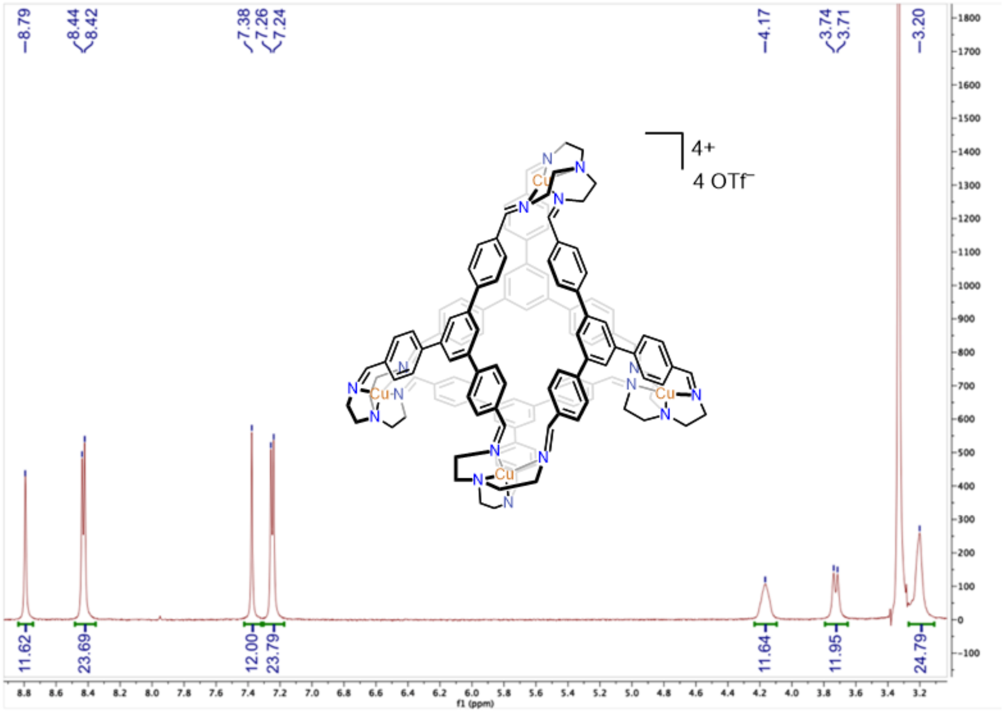

Figure S26. ^{1}H NMR (500 MHz, 23 ºC) spectrum of **1-Cu** in DMSO-d_6. The unpicked singlet at 3.33 ppm corresponds to water.

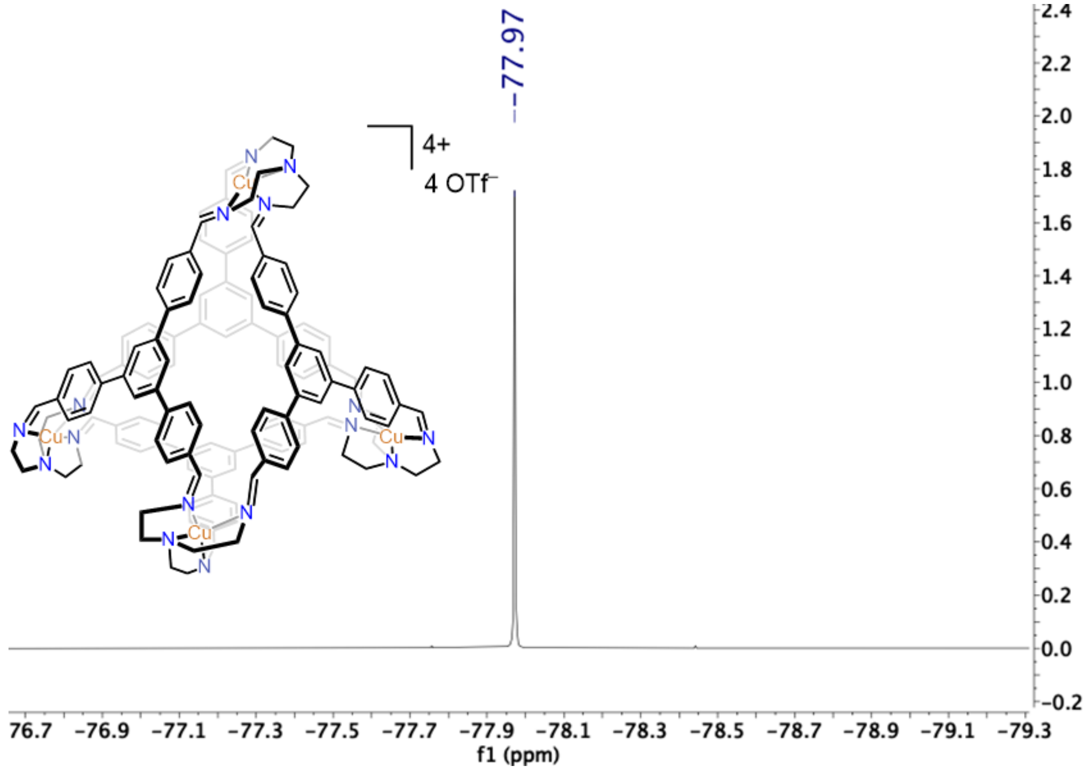

Figure S27. ^{19}F NMR (470 MHz, 23 ºC) spectrum of **1-Cu** in DMSO-d$_6$.

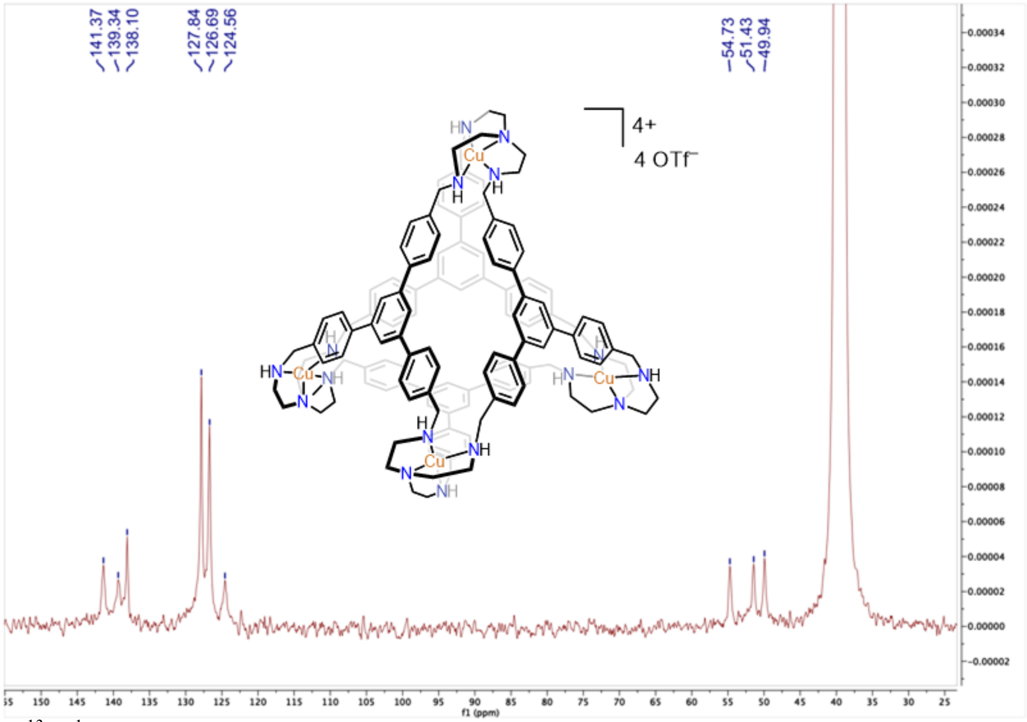

Figure S28. $^{13}C\{^1H\}$ NMR (101 MHz, 23 ºC) spectrum of **2-Cu** in DMSO-d_6. The unpicked multiplet centered at 39.52 ppm corresponds to the DMSO-d_6 solvent residual.

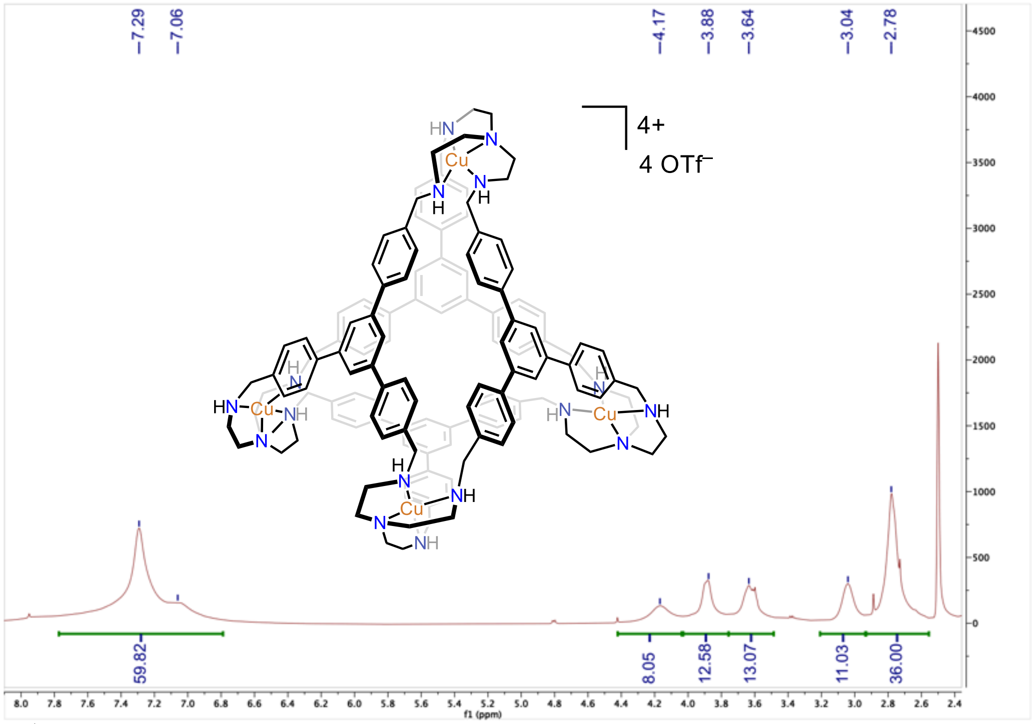

Figure S29. ^{1}H NMR (500 MHz, 23 ºC) spectrum of **2-Cu** in DMSO-d_6. The unpicked singlet at 2,50 ppm corresponds to the DMSO-d_6 solvent residual.

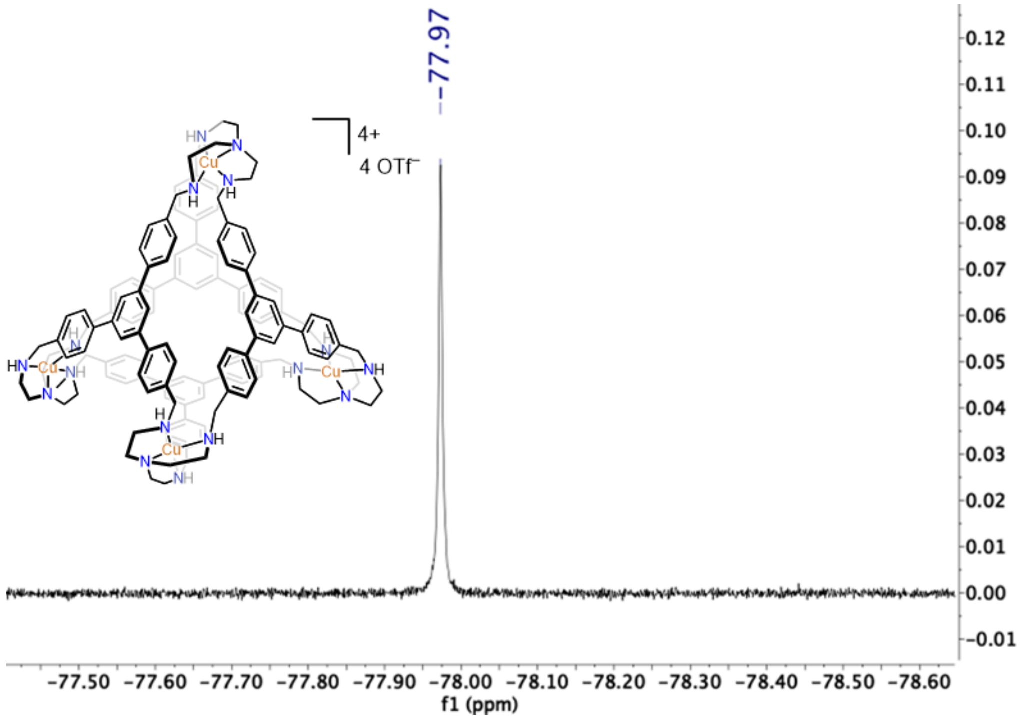

Figure S30. ^{19}F NMR (470 MHz, 23 ºC) spectrum of **2-Cu** in DMSO-d_6.

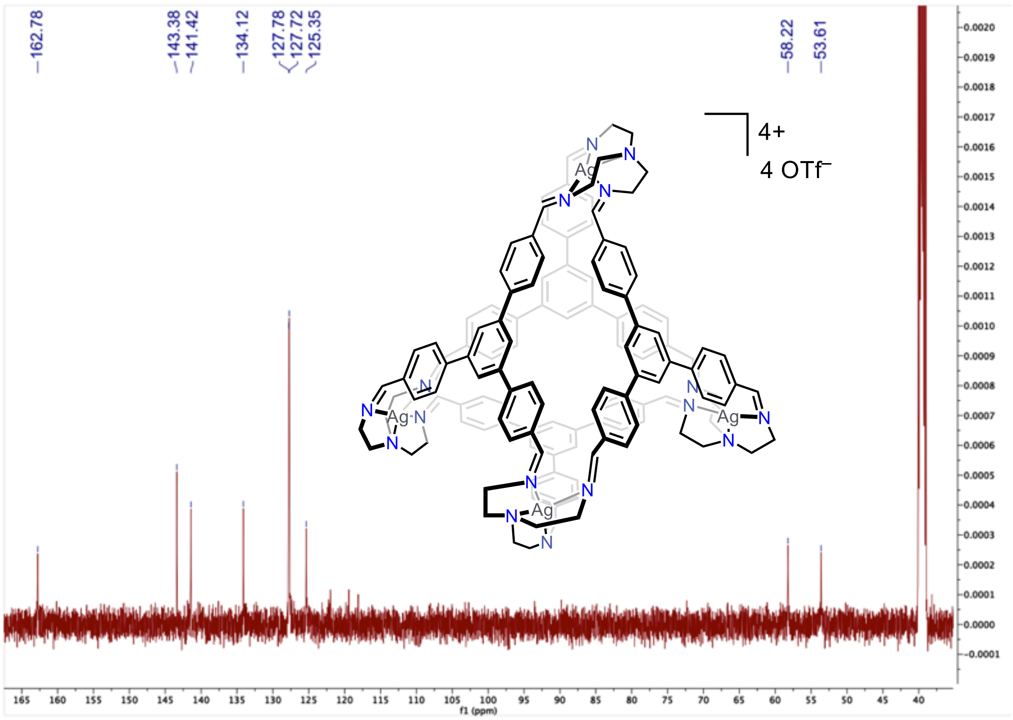

Figure S31. $^{13}C\{^{1}H\}$ NMR (126 MHz, 23 ºC) spectrum of **1-Ag** in DMSO-d_6. The unpicked multiplet centered at 39.52 ppm corresponds to the DMSO-d_6 solvent residual.

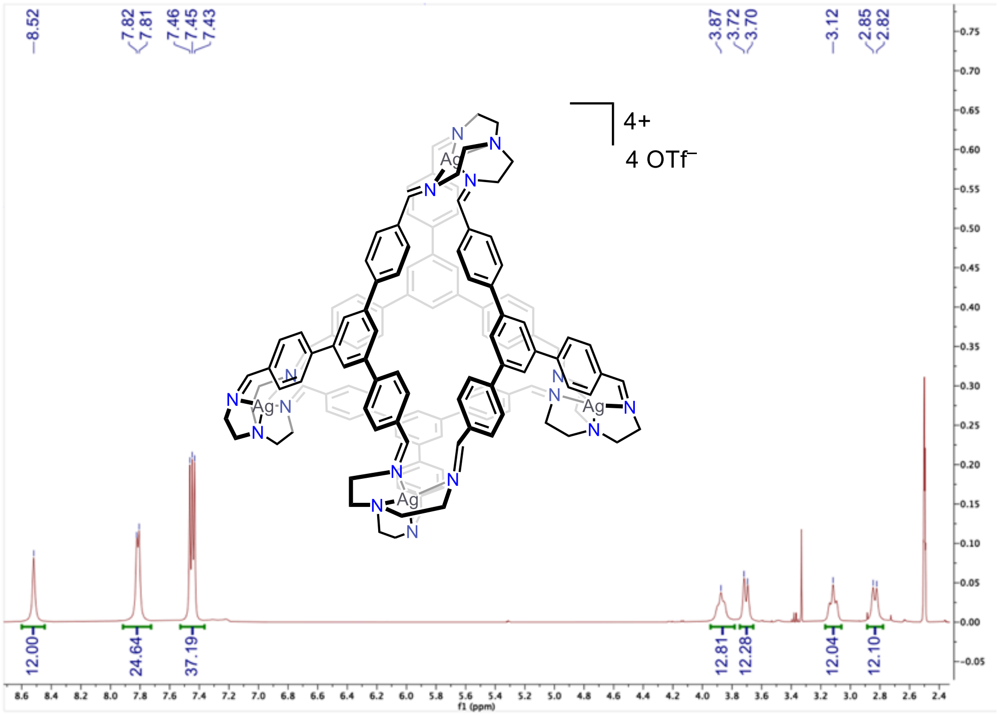

Figure S32. ^{1}H NMR (500 MHz, 23 ºC) spectrum of **1-Ag** in DMSO-d_6. The unpicked singlet centered at 2.50 ppm corresponds to the DMSO-d_6 solvent residual. The unpicked singlet centered at 3.33 pm corresponds to water.

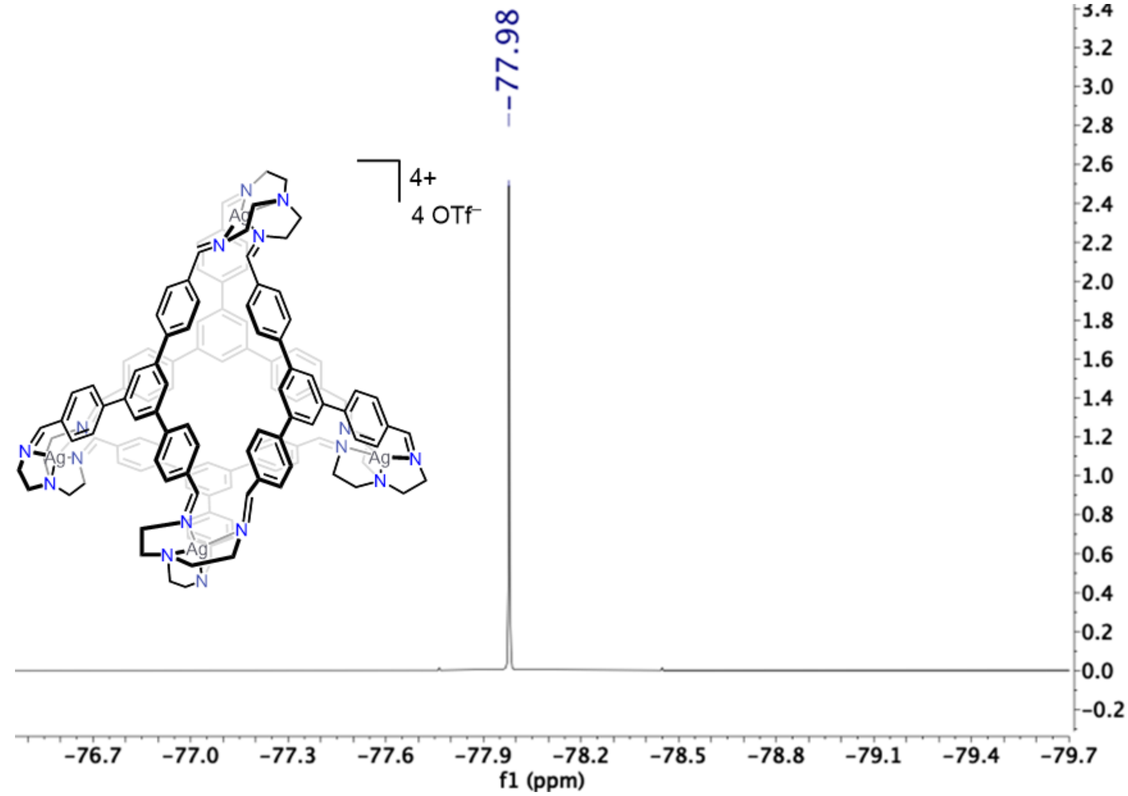

Figure S33. ^{19}F NMR (470 MHz, 23 ºC) spectrum of **1-Ag** in DMSO-d_6.

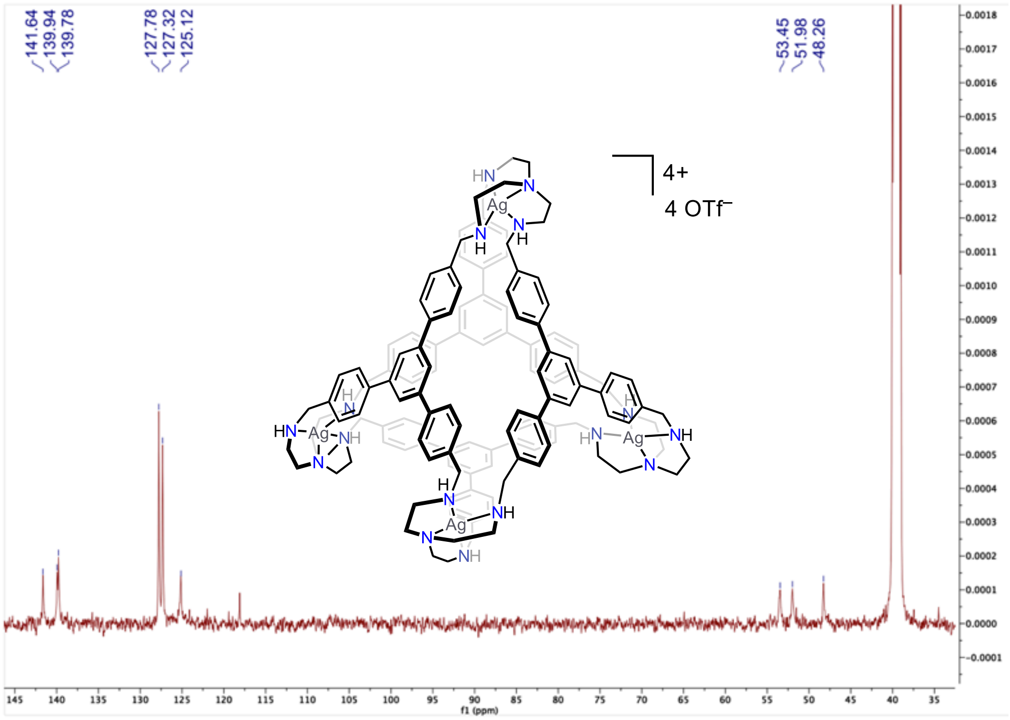

Figure S34. $^{13}C\{^{1}H\}$ NMR (126 MHz, 23 ºC) spectrum of **2-Ag** in DMSO-d_6. The unpicked multiplet centered at 39.52 ppm corresponds to the DMSO-d_6 solvent residual.

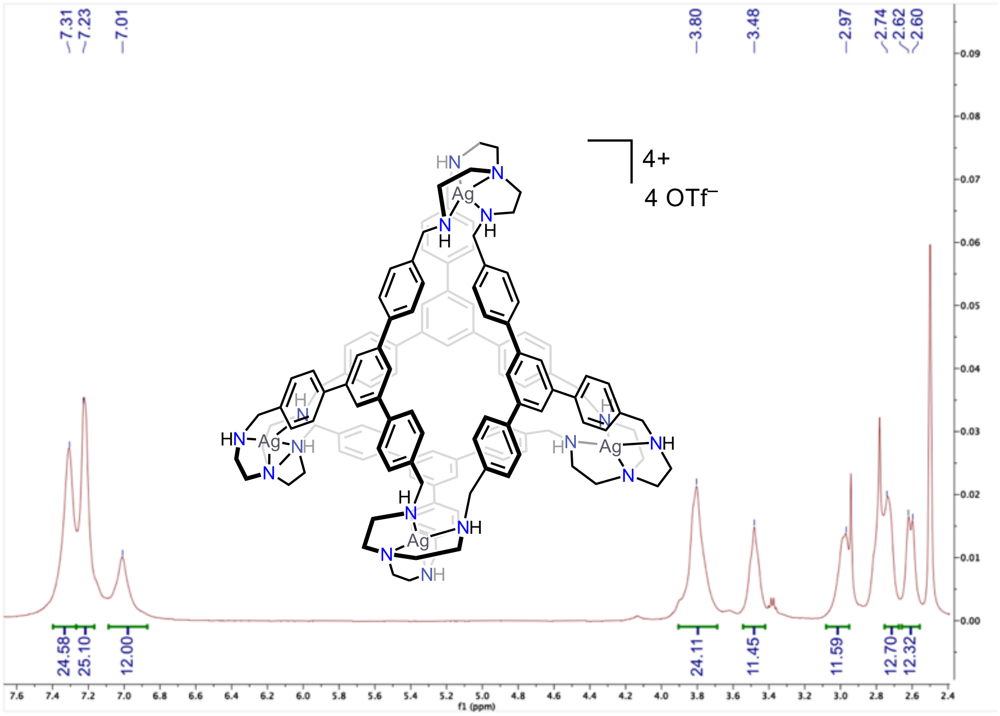

Figure S35. ^{1}H NMR (500 MHz, 23 ºC) spectrum of **2-Ag** in DMSO-d_6. The unpicked singlet centered at 2.50 ppm corresponds to the DMSO-d_6 solvent residual.

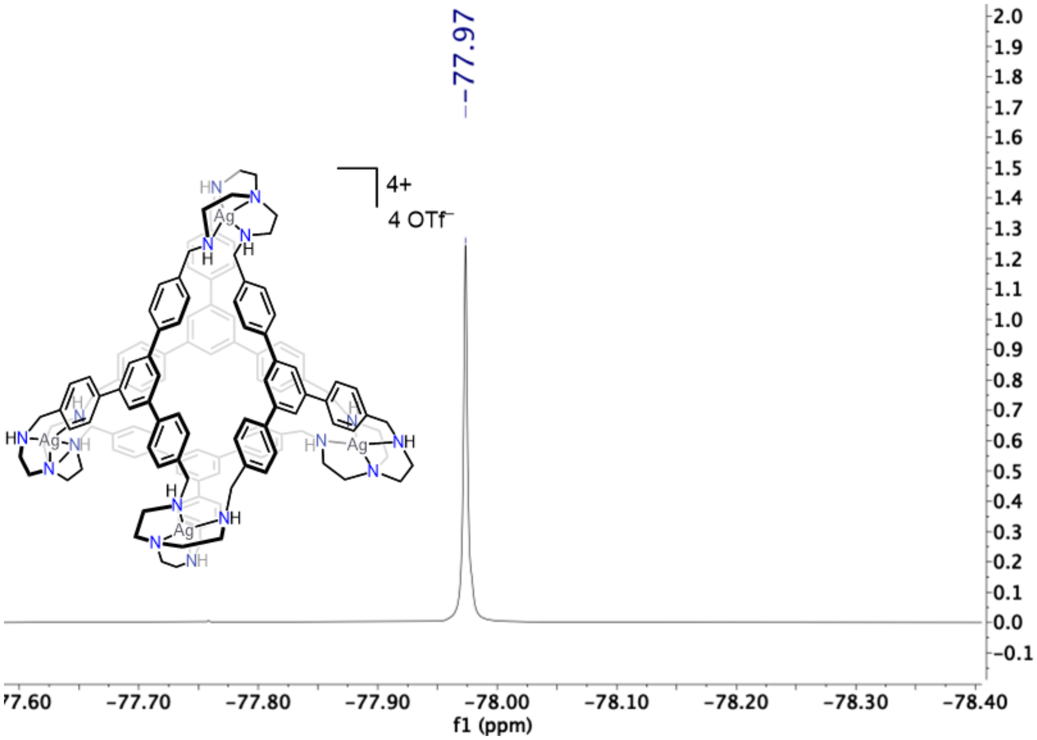

Figure S36. ^{19}F NMR (470 MHz, 23 ºC) spectrum of **2-Ag** in DMSO-d_6.

6. Thermogravimetric Analysis Measurements

Thermogravimetric decomposition measurements were performed on a TA instruments TGA55 under an atmosphere of N_2 gas. The material was transferred to a pre-weighed disposable aluminium pan, which was loaded onto a platinum carrier pan. This was loaded into the instrument.

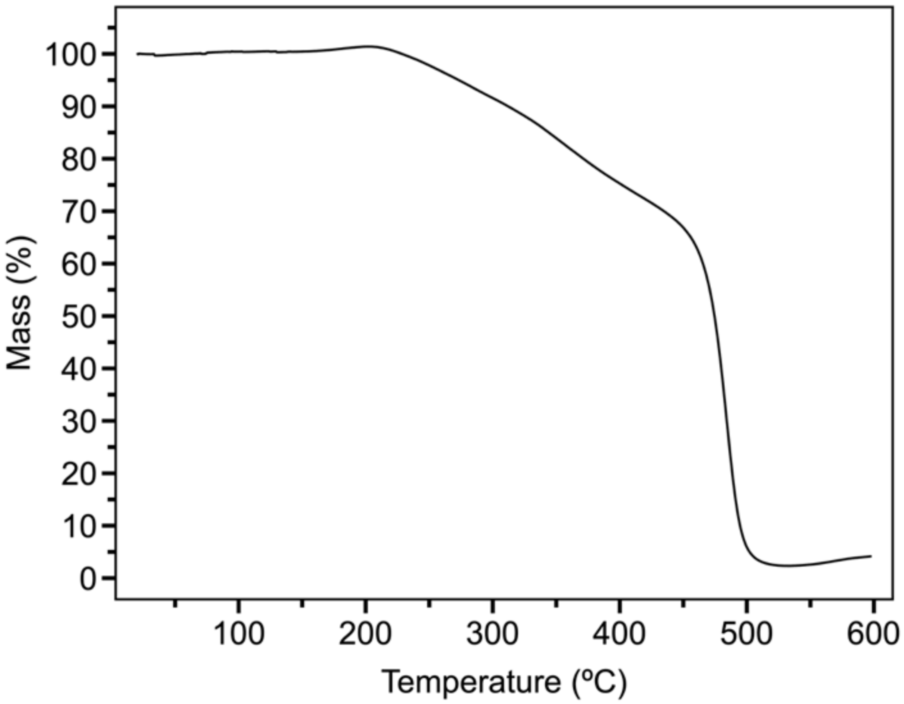

Figure S37. Thermogravimetric analysis (TGA) of **1**. The temperature was ramped at a rate of 1.0 ºC/min from 25 ºC to 600 ºC.

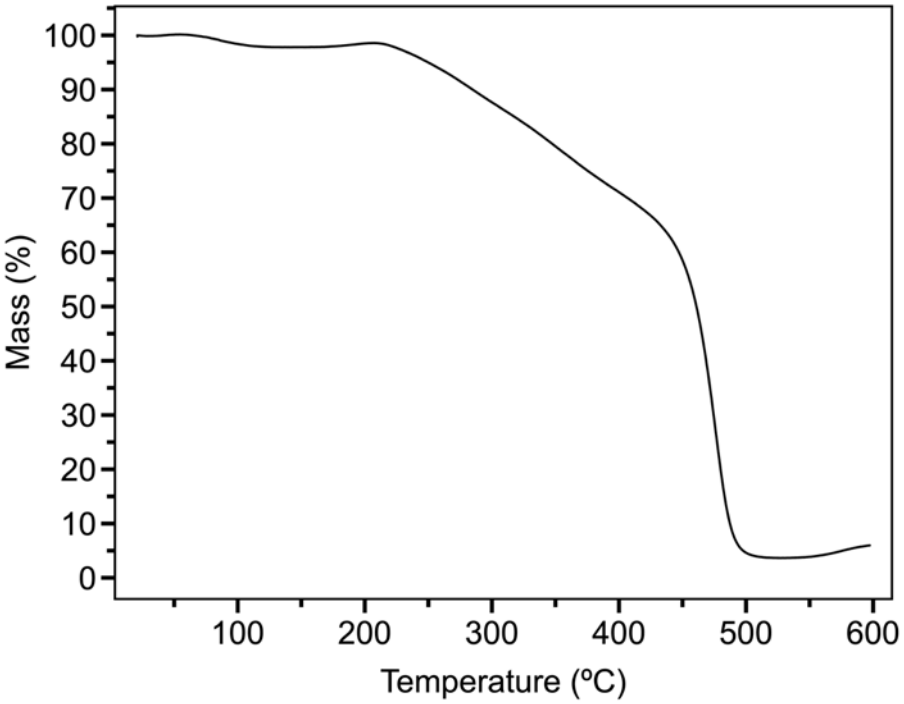

Figure S38. TGA of **2**. The temperature was ramped at a rate of 1.0 ºC/min from 25 ºC to 600 ºC.

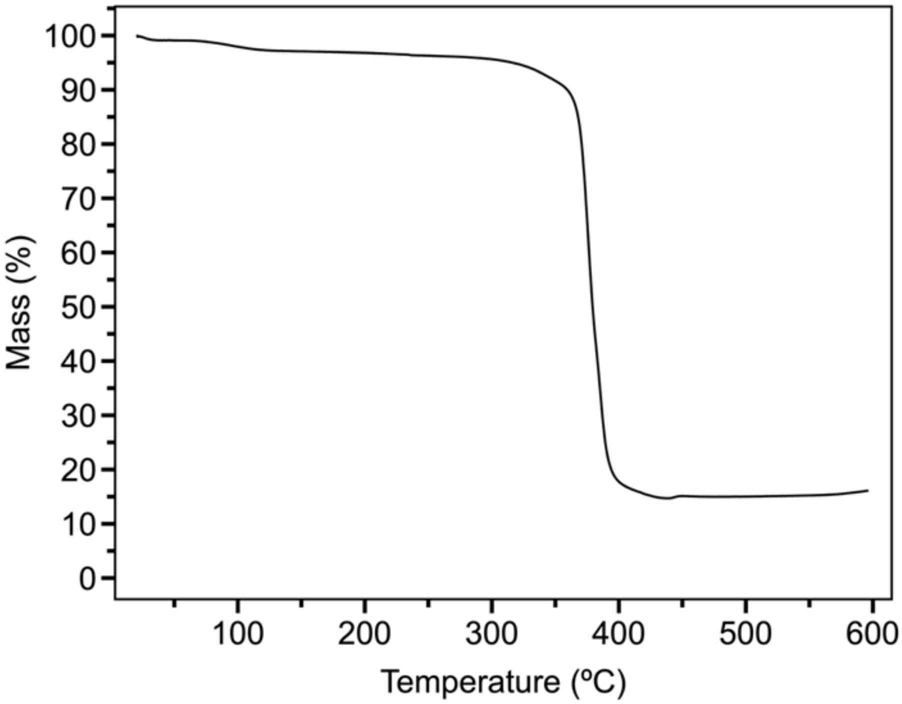

Figure S39. TGA of **1-Cu**. The temperature was ramped at a rate of 5.0 ºC/min from 25 ºC to 600 ºC

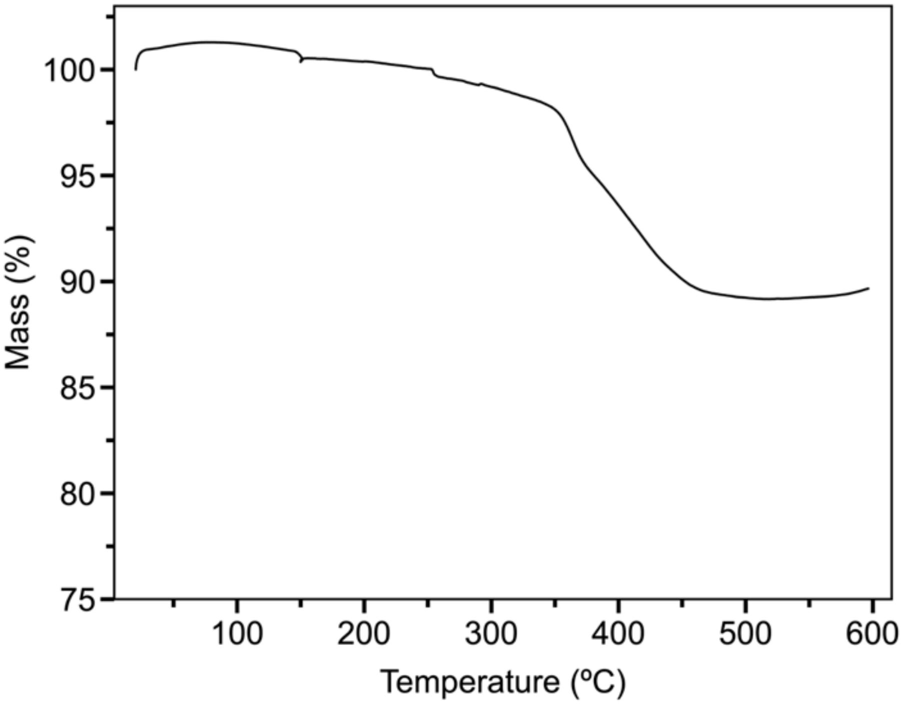

Figure S40. TGA of **2-Cu**. The temperature was ramped at a rate of 10.0 ºC/min from 25 ºC to 150 ºC. The heat was held isothermally for 120.0 minutes, and then the temperature was ramped at a rate of 5.0 ºC/min from 150 ºC to 600 ºC.

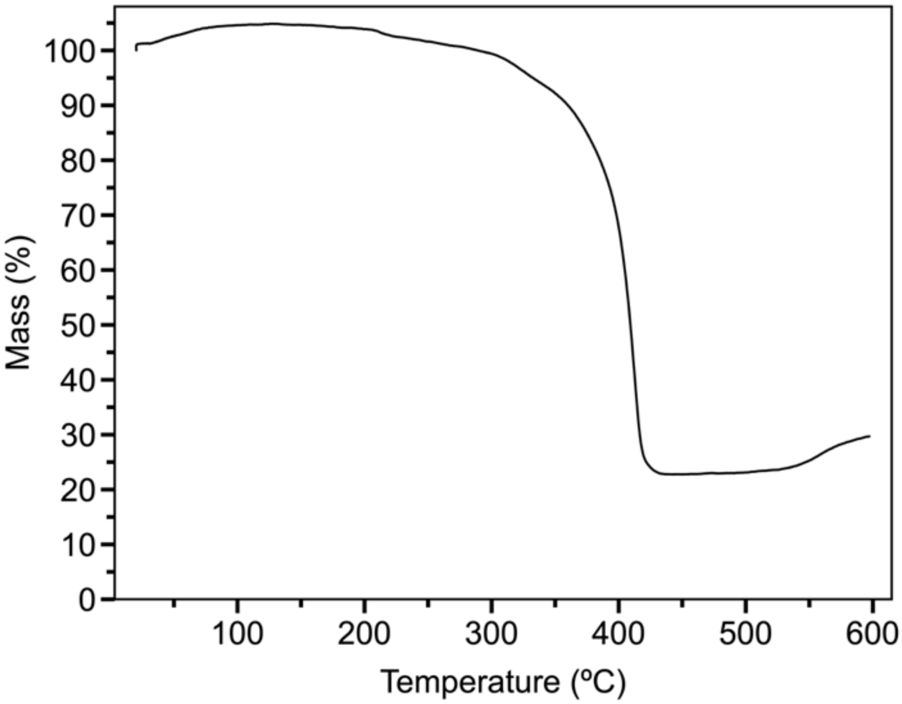

Figure S41. TGA of **1-Ag**. The temperature was ramped at a rate of 1.0 ºC/min from 25 ºC to 600 ºC.

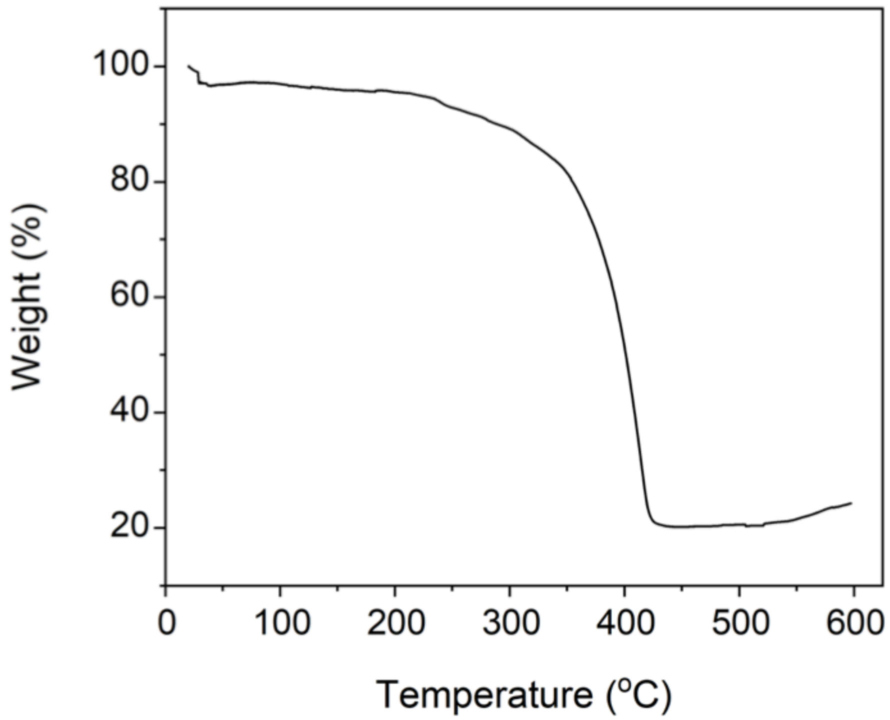

Figure S42. TGA of **2-Ag**. The temperature was ramped at a rate of 1.0 ºC/min from 25 ºC to 600 ºC.

7. References

1. Myers, A. L.; Prausnitz, J. M. Thermodynamics of Mixed-Gas Adsorption. *AIChE J.* **1965**, *11*, 121–127.

2. Rouquerol, J.; Llewellyn, P.; Rouquerol, F. Is the BET Equation Applicable to Microporous Adsorbents? In *Characterization of Porous Solids VII, Studies in Surface Science and Catalysis, Vol. 160*. Llewellyn, P.; Rodriguez-Reinoso, F.; Rouquerol, J.; Seaton, N., Eds.; Elsevier: Amsterdam and Oxford, **2007**, pp 49–56.

3. *CrysAlisPro*, version 171.42.64a; Rigaku Corporation: Oxford, UK, 2022

4. Sheldrick, G. M. SHELXT – Integrated Space-Group and Crystal-Structure Determination. *Acta. Crystallogr.* **2015**, *A71*, 3-8.

5. Sheldrick, G. M. Crystal Structure Refinement with SHELXL. *Acta. Crystallogr.* **2015**, *C71*, 3-8.

6. Spek, A. L. PLATON SQUEEZE: A Tool For the Calculation of the Disordered Solvent Contribution to the Calculated Structure Factors. *Acta Crystallogr.* **2015**, *C71*, 9–18.
